# Mandible mechanical properties and composition of the larval *Glossosoma boltoni* (Trichoptera, Insecta)

**DOI:** 10.1038/s41598-024-55211-5

**Published:** 2024-02-26

**Authors:** Wencke Krings, Patrick Below, Stanislav N. Gorb

**Affiliations:** 1 Department of Electron Microscopy, Institute of Cell and Systems Biology of Animals, Universität Hamburg, Martin-Luther-King-Platz 3, 20146 Hamburg, Germany; 2 Department of Cariology, Endodontology and Periodontology, Universität Leipzig, Liebigstraße 12, 04103 Leipzig, Germany; 3 Department of Mammalogy and Palaeoanthropology, Leibniz Institute for the Analysis of Biodiversity Change, Martin-Luther-King-Platz 3, 20146 Hamburg, Germany; 4 Department of Functional Morphology and Biomechanics, Zoological Institute, Christian-Albrechts-Universität zu Kiel, Am Botanischen Garten 1-9, 24118 Kiel, Germany

**Keywords:** Cuticle, Elemental composition, Mechanical properties, Feeding, Wear, Structural biology, Biomechanics

## Abstract

Insect feeding structures, such as mandibles, interact with the ingesta (food or/and substrate) and can be adapted in morphology, composition of material and mechanical properties. The foraging on abrasive ingesta, as on algae covering rocks, is particularly challenging because the mandibles will be prone to wear and structural failure, thus suggesting the presence of mandibular adaptations to accompany this feeding behavior. Adaptations to this are well studied in the mouthparts of molluscs and sea urchins, but for insects there are large gaps in our knowledge. In this study, we investigated the mandibles of a grazing insect, the larvae of the trichopteran *Glossosoma boltoni*. Using scanning electron microscopy, wear was documented on the mandibles. The highest degree was identified on the medial surface of the sharp mandible tip. Using nanoindentation, the mechanical properties, such as hardness and Young’s modulus, of the medial and lateral mandible cuticles were tested. We found, that the medial cuticle of the tip was significantly softer and more flexible than the lateral one. These findings indicate that a self-sharpening mechanism is present in the mandibles of this species, since the softer medial cuticle is probably abraded faster than the harder lateral one, leading to sharp mandible tips. To investigate the origins of these properties, we visualized the degree of tanning by confocal laser scanning microscopy. The autofluorescence signal related to the mechanical property gradients. The presence of transition and alkaline earth metals by energy dispersive X-ray spectroscopy was also tested. We found Ca, Cl, Cu, Fe, K, Mg, Mn, P, S, Si, and Zn in the cuticle, but the content was very low and did not correlate with the mechanical property values.

## Introduction

Grazing animals, such as molluscs, sea urchins, catfish and tadpoles remove biofilms by pressing their highly specialized mouthparts to the rough substrate surface (see^1–7^). Through the persistent interaction with abrasive substrates, structures are prone to experience failure or high degrees of wear. Both can be reduced by a high bending capacity of the interacting structures, as found in e.g., molluscan^8,9^ or catfish teeth^2,10–12^, or by increasing the hardness of the interacting surface by e.g., incorporating high proportions of inorganics or increasing fibre densities, as found in e.g., sea urchins and molluscan teeth^3,5,6,13,14^.

In some grazing insects, e.g., taxa belonging to Ephemeroptera, Plecoptera, Coleoptera, Diptera, or Trichoptera, mandibles function as excavators removing the algae from stones. Additionally, they can carry setae functioning as brushes, brooms, or combs, which gather the loosened algae afterwards^2,10,11,15–19^. In some insects, the mandibles are even capable of drilling through rocks, as determined in some mayfly larvae^20^. This interaction results in wear and tear on mandibles, which could reduce the efficiency of feeding^2,11^. Potential mechanisms to retain the functionality could be self-healing mechanisms, which were previously reported for insect cuticle^21–23^. As documented in the larvae of the trichopteran *Glossosoma boltoni* Curtis, 1834, (Glossosomatidae) grazing activity can also be facilitated by elongated blades of the mandibles, which become abraded during feeding leaving a sharp edge^2,10,11^. Additionally, the mechanical properties of the mandibles could support failure- and wear-prevention.

Only a few studies have targeted the mechanical properties and their origins in insect and arthropod mouthparts (see^24–27^). In general, the mechanical properties have their origin in the degree of sclerotisation and the distribution of proteins as e.g., resilin. Additionally, some inorganic compounds in insect cuticle provide more hardness and stiffness (^28–30^; see reviews^31,32^). Transition metals such as Cu, Fe, Mn and Zn are involved in the formation of complex molecules by binding strongly to the biopolymers and increase the cross-linking density^31,33–37^. Additionally, alkaline earth metals, such as Ca and Mg, which could indicate biomineralization, have been previously detected in insect cuticle^27,38–41^.

With regard to the material composition and corresponding mechanical properties facilitating the grazing activity in insect mandibles, there are large gaps in our knowledge. To provide insight into mechanisms contributing to the wear- and failure-prevention in insect cuticle, we here study the mandible of the larvae of *Glossosoma boltoni*, which scrape epilithic algae and detritus from stones^42^. It belongs to the holometabolous order Trichoptera, which contains about 14,500 extant species^43^. Larval mandibles of this species have been previously well studied with regard to morphology^2,10,11^. *Glossosoma boltoni* can be found in large streams and small to medium-sized rivers^44–48^ throughout Europe, excluding Scandinavia and the southern Balkan, eastwards to north- and northwest-Russia. The larvae are inhabitants of the upper side of stones and are not found on muddy or sandy substratum^47^. The adults are present from April to October^47^.

In the present study, the mandibles were first observed using scanning electron microscopy, to identify the regions affected from wear. Then, the mechanical properties, such as hardness *H* and Young’s modulus *E* of the mandible cuticle, were tested using nanoindentation. Our results on the distribution of material properties indicate that the self-sharpening mechanism is present in the mandibles of this species. To determine the origins of mechanical properties, confocal laser scanning microscopy (CLSM) was applied to study the cuticle autofluorescence, which can provide information on the distribution of certain constituents and the degree of tanning. Additionally, elemental analyses using energy dispersive X-ray spectroscopy (EDX, EDS) were performed on the same sites that were tested by nanoindentation, to identify inorganic content of the cuticle. We found that transition and alkaline earth metals are present in low proportions and do not relate to the mechanical property values. This is in contrast to the autofluorescence signal, which is well related to the mechanical properties. This data set is new and shall provide deeper insight into the biomechanics of insect mouthparts.

## Materials and methods

### Specimens and preparation

Individuals of *Glossosoma boltoni* were collected by Prof. A. Thienemann at Plön, Germany, in 1930, and fixed in 70% EtOH (inventory number: 210, Leibniz Institute for the Analysis of Biodiversity Change, former Zoological Museum Hamburg). To perform this study, ten specimens of the ultimate larval instar were dissected. The material used is old, but we think that the material can still be used since the here presented results are part of a larger study on trichopteran mandibles. We tested the mandibles of six species; most of the material used was older, but we also studied samples that were preserved for only few years in 70% EtOH. The results from our EDX and nanoindentation measurements on *G. boltoni* are within the results from the fresher specimens. In these latter, no relationship between the elements and the mechanical properties could be detected. In addition, the autofluorescence signal received from *G. boltoni* was similarly strong to the signal received from the fresher cuticle. Surely, the drying and embedding procedure alters material properties of cuticle to some extent, but structures with a high 3dimensionality cannot be measured by nanoindentation and EDX.

### Light microscopy

All specimens were first documented by light microscopy, employing a Keyence Digital Microscope VHX-7000 (KEYENCE, Neu-Isenburg, Germany) equipped with automatic stacking software.

### Scanning electron microscopy (SEM)

Two heads with the first thoracic segments were deposited in small tubes that were filled with 70% EtOH and cleaned by a short ultrasonic bath for 20 s. The morphology was documented in SEM. First, heads were mounted on SEM specimen holders by double-sided adhesive carbon tape. After drying at room temperature for 30 min, samples were then sputter-coated with platinum (5 nm layer) and visualized with the Zeiss LEO 1525 (One Zeiss Drive, Thornwood, NY, USA). Afterwards, samples were rewetted by 70% EtOH. Then, the heads were dissected, the four mandibles carefully extracted, cleaned by a short ultrasonic bath for 20 s, to remove the previous sputter-coating, arranged on SEM sample holders, sputter-coated and visualized with the Zeiss Leo again (15 kV, magnifications were altered between the images).

### Staining

To test whether the localities on the mandibles, which appeared worn under SEM, really experienced abrasion of the epicuticle (which reduces the intrusion of fluids into the cuticle), two mandibles of one additional specimen were stained. For this purpose, mandibles were deposited in toluidine blue (Sigma-Aldrich, St. Louis, MO, USA; 1 g in 100 ml 70% EtOH) for 2 min. The toluidine blue binds strongly to chitin and can this visualize areas without epicuticle. Afterwards, the whole mounts were documented using light microscopy.

### Confocal laser scanning microscopy (CLSM)

For this step, three mandibles of two additional specimens were cleaned by a short ultrasonic bath for 20 s. Before visualization of the autofluorescence, each mandible was documented from both sides using light microscopy, to identify potential pigmentation of cuticle. Two unbroken mandibles (one in ventral and one in dorsal view) and one mandible with cut-off tip (in medial view) were arranged on object glass slides, following the procedure of^49^. Each mandible was surrounded by a stack of reinforcement rings, filled with glycerine (greater than or equal to 99.5%, free of water, Carl Roth GmbH & Co. KG, Karlsruhe, Germany) and covered with a cover slip. Following previous protocol^41,49^, samples were visualized with a Zeiss LSM 700 confocal laser scanning microscope (Carl Zeiss Microscopy GmbH, Jena, Germany), equipped with four stable solid-state lasers with wavelengths of 405 nm, 488 nm, 555 nm, and 639 nm. Bandpass or longpass emission filters (420–480 nm, greater than or equal to 490 nm, greater than or equal to 560 nm, or greater than or equal to 640 nm) were used. After scanning, images of autofluorescence were superimposed (with maximum intensity projection) using the software Zeiss Efficient Navigation (Zen) (Carl Zeiss MicroImaging GmbH). Finally, the color blue was assigned to the autofluorescence signal received from the laser with wavelength 405 nm, green to 488 nm, red (50% saturation) to 555 nm and red (50% saturation) to 639 nm.

### Energy dispersive X-ray spectroscopy (EDX)

With EDX we tested two mandibles (one left mandible of one specimen and one right mandible of another specimen to compare for differences between individuals and between right and left side). The same localities of the counter mandibles from the same specimens were later tested by nanoindentation.

For this purpose, the two clean (20 s in ultrasonic bath) and dry mandibles were attached to glass object slides by double-sided adhesive carbon tape, following previous protocols^9,41,50^. Then, each mandible was surrounded by a small metallic ring. This ring was afterwards filled with epoxy resin (Reckli Epoxy WST, RECKLI GmbH, Herne, Germany) to cover the mandibles completely. Polymerization lasted for three days at room temperature.

Glass object slide and carbon tape were removed and each sample polished with different sand papers until a region of interest was on display. Since the different regions of interest (e.g., ventral and dorsal condyle) are at different levels of the sample, a sample was always polished until one target region was on display, then EDX and nanoindentation were performed. Afterwards the sample was polished until the next target region was on display. Then each step of the protocol was repeated (see below) and the next regions were tested.

To prepare samples for the EDX, the surface was, after polishing, smoothened with aluminium oxide polishing powder suspension of 0.3 μm grainsize (PRESI GmbH, Hagen, Germany) on a polishing machine (Minitech 233/333, PRESI GmbH, Hagen, Germany) to receive a plain sample surface. Before measurements, embedded mandibles were cleaned in an ultrasonic bath for 5 min to remove the polishing powder. We used sections of the mandibles to test the exocuticle by nanoindentation and EDX, but not the thin epicuticle.

Samples were mounted on SEM sample holders and sputter-coated with platinum (5 nm layer). We chose platinum for coating to have one element that could be used as marker to check if each individual EDX measurement was correct and not corrupted by e.g., surface roughness. For this, we measured later 20 areas of pure epoxy to receive values on the Pt content (mean ± SD; 0.14 ± 0.02 atomic %). After all analyses were completed, we excluded the measurements (N = 3) with very high proportions of Pt (e.g., more than 5 atomic %).

Elemental composition was determined with the SEM Zeiss LEO 1525 equipped with an Octane Silicon Drift Detector (SDD) (micro analyses system TEAM, EDAX Inc., New Jersey, USA). For each sample, the same settings were used (i.e. an acceleration voltage of 20 kV, working distance, lens opening, etc.). Before analysis, the detector was calibrated with copper. We did not perform mappings, but instead analysed small areas of the cuticle sections.

We detected Al (aluminium), C (carbon), Ca (calcium), Cl (chlorine), Cu (copper), Fe (iron), H (hydrogen), K (potassium), Mg (magnesium), Mn (manganese), N (nitrogen), O (oxygen), P (phosphorus), Pt (platinum), S (sulphur), Si (silicon) and Zn (zinc) in our samples and measured their proportions. Na was detected in only two point measurements and is probably an artifact. We displayed it in the figures, but did not discuss Na in the manuscript.

We also did not discuss the following elements, as they are the elemental basis of chitin and proteins (H, C, N, O), the coating (Pt), or the polishing powder (Al, O). For test purposes, we performed 10 EDX point measurements on the epoxy, but could not detect Si (which could be part of the sandpaper), or of other elements that we further discuss—which shows, that these elements are not an artifact of embedding and polishing, but are part of the mandibles.

The single peak of P overlaps with the one of Pt. Due of this, the software could not discriminate between these two elements and P content could not be reliably determined. Therefore, P and Pt were discussed together (P + Pt). As mentioned above, we measured 20 areas of pure epoxy to receive values on the Pt content (mean ± SD; 0.14 ± 0.02 atomic %) to further estimate the proportions of P.

Overall, we tested 141 small (1–2 × 1–2 µm) areas (on the lateral surface: 40; on the tip: 41; on the medial surface: 40; on the condyles: 20).

### Nanoindentation

Nanoindentation was performed on the two counter mandibles from the same specimens that were studied by EDX. Additionally, to increase the sample size, six mandibles of three more specimens were used for mechanical property tests (thus, overall four individuals were studied). Samples for nanoindentation were prepared following the same protocol as used for EDX: mandibles were embedded in epoxy, samples polished and smoothened (for detailed protocol, see^9,50^).

Samples were then attached onto the nanoindenter sample holder. Indentation was performed with a nanoindenter SA2 (MTS Nano Instruments, Oak Ridge, Tennessee, USA), equipped with a Berkovich indenter tip and a dynamic contact module (DCM) head. The mechanical properties hardness (*H*) and Young’s modulus (*E*) were determined from force-distance curves by applying the continuous stiffness mode. *E* and *H* were determined at penetration depths of 800–1000 nm. For each site indented, we received ~ 30 values obtained at different indentation depths, which were averaged to receive one *H* and one *E* mean value per indent. All tests were performed under normal room conditions (relative humidity 28–30%, temperature 22–24 °C) and each indent and corresponding curve were both manually controlled. After this, each sample was smoothened and polished until the next target region was on display.

Overall, 208 localities from eight mandibles were tested by nanoindentation*:* 8 medial surfaces of the tip; 8 lateral surfaces of the tip; 88 measurements along the medial surfaces of the mandibles; 88 measurements along the lateral surfaces of the mandibles; and one indent per condyle and per specimens (8 measurements for the ventral and 8 for the dorsal condyles).

### Statistical analyses

All statistical analyses were performed with JMP Pro, Version 14 (SAS Institute Inc., Cary, NC, 1989–2007). Mean values and standard deviations were calculated and Shapiro–Wilk-*W*-tests for testing of normality was conducted. As the data was non-normally distributed, a Kruskal–Wallis test, followed by pairwise comparison with Wilcoxon method, was carried out. With this software, correlation coefficients were calculated.

## Results

### Morphology and wear

**Figure 1:**
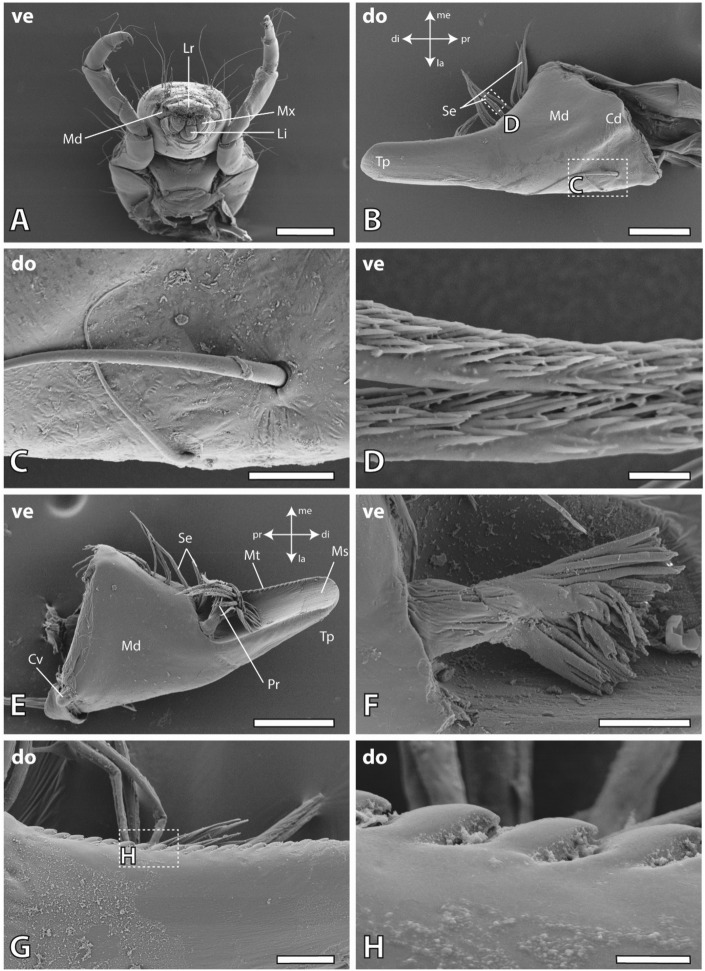
SEM images of the head and mandibles of larval *Glossosoma boltoni*. (**A**) Ventral view on the head and the first thoracic segment of one specimen. (**B**) Dorsal view on the left mandible. (**C**) Magnification of the trichoid sensilla. (**D**) Magnification of the setae surfaces. (**E**) Ventral view on the left mandible with wear on the medial surface of the tip. (**F**) Magnification of the prostheca. (**G**,**H**) Mandibular teeth on the dorsal edge of the mandible with magnification (**H**). *Cd* dorsal condyle, *Cv* ventral condyle, *di* distal, *la* lateral, *Li* labium, *Lr* labrum, *me* medial, *Md* mandible, *Ms* medial surface, *Mt* mandibular tooth, *Mx* maxilla, *pr* proximal, *Pr* prostheca, *Se* setae, *Tp* tip, *ve* ventral. Scale bars: (**A**) 400 µm; (**B**,**E**) 80 µm; (**C**,**F**,**G**) 20 µm; (**D**,**H**) 4 µm.

**Figure 2:**
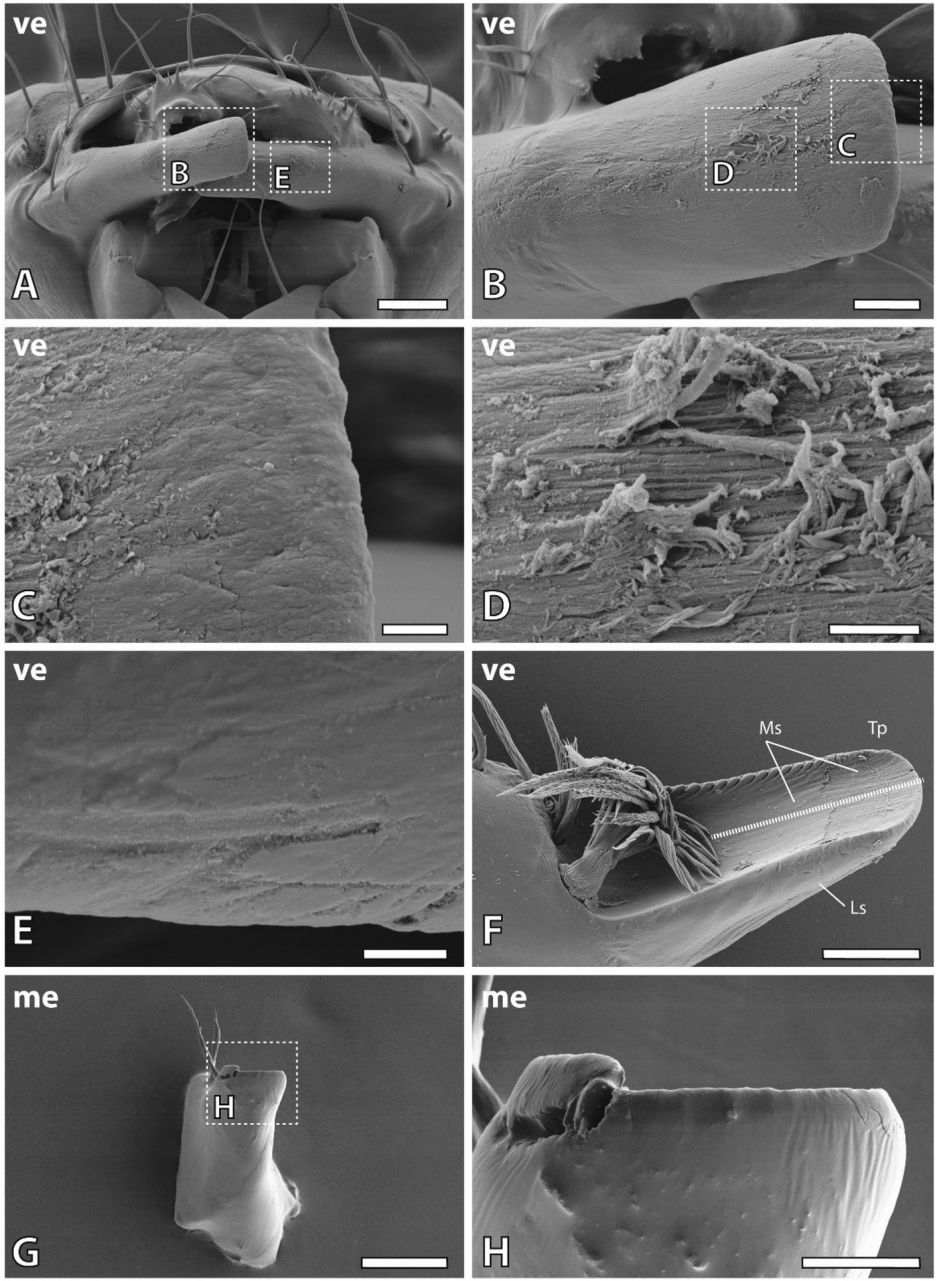
SEM images of wear on the mandibles of larval *Glossosoma boltoni*. (**A**) Ventral view on the head, showing the interaction between both mandibles. (**B**–**D**) Ventral view on the right mandible (**B**) with magnification of the scratches on the tip (**C**) and the wear on the lateral surface exposing the chitin fibres (**D**). (**E**) Magnification of scratches on lateral surface of the left mandible. (**F**) Ventral view on the left mandible with high degrees of wear on the medial surface of the tip exposing the chitin fibres. The dashed line represents the locality of the section (on the embedded sample) to test the mechanical properties and the elemental composition of the medial and lateral mandible surface. (**G**,**H**) Medial view on the right mandible at a high magnification (**H**) showing the sharp cutting edge of the mandible tip. *di* distal, *do* dorsal, *la* lateral, *Ls* lateral surface (the surface of the mandible facing towards lateral), *me* medial, *Ms* medial surface (the surface of the mandible facing towards medial), *pr* proximal, *Tp* tip, *ve* ventral. Scale bars: (**A**,**G**) 80 µm; (**B**,**H**) 20 µm; (**C**–**E**) 4 µm, (**F**) 40 µm.

In *Glossosoma boltoni*, the mandibles were partly covered by the labrum and the maxillae (Fig. 1A). The lateral surface (for the orientation see Fig. 1) of one mandible (either the left or the right one) interacted with the medial surface of the other one (either the right one or the left one), which became visible, when the labrum and the maxillae were removed (Fig. 2A). Each mandible carried two trichoid sensilla on their lateral side (Fig. 1B,C) and multiple serrate setae (Fig. 1B,D) as well as a broom-like prostheca on its medial side (Fig. 1E,F). The dorsal edge of the mandibles possessed multiple mandibular teeth which were pointed towards medial (Fig. 1G,H).

High wear was detected on the medial surface of the tip on the left and the right mandible (Figs. 1E and 2F). We documented scratches and wear on the lateral surface of the mandibles as well, the abrasion degree was however lower (Fig. 2A–E). The very tips of the mandibles had a sharp edge, which could be determined when the mandibles were documented in medial view (Fig. 2G,H).

**Figure 3:**
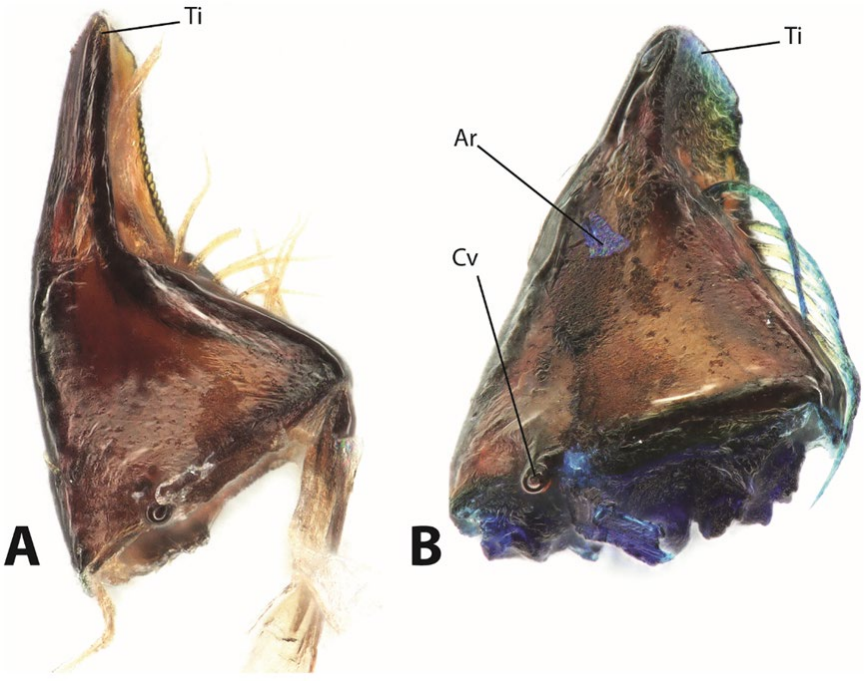
(**A**) Light microscopy image of an unstained mandible. (**B**) Mandible stained with toluidine blue. The setae, internal tissues and the mandible tip are blue. *Ar* artifact, a loose piece of tissue, *Cv* ventral condyle, *Ti* tip.

After the mandibles were stained by toluidine blue, the surrounding tissues, sensilla, prostheca and setae appeared slightly bluish (Fig. 3). Additionally, the medial surface of the mandible tip appeared blue, which was in contrast to the rest of the mandible cuticle. This suggests, that the epicuticle of the tip was not present and the stain directly entered the exocuticle.

### Autofluorescence signals

**Figure 4:**
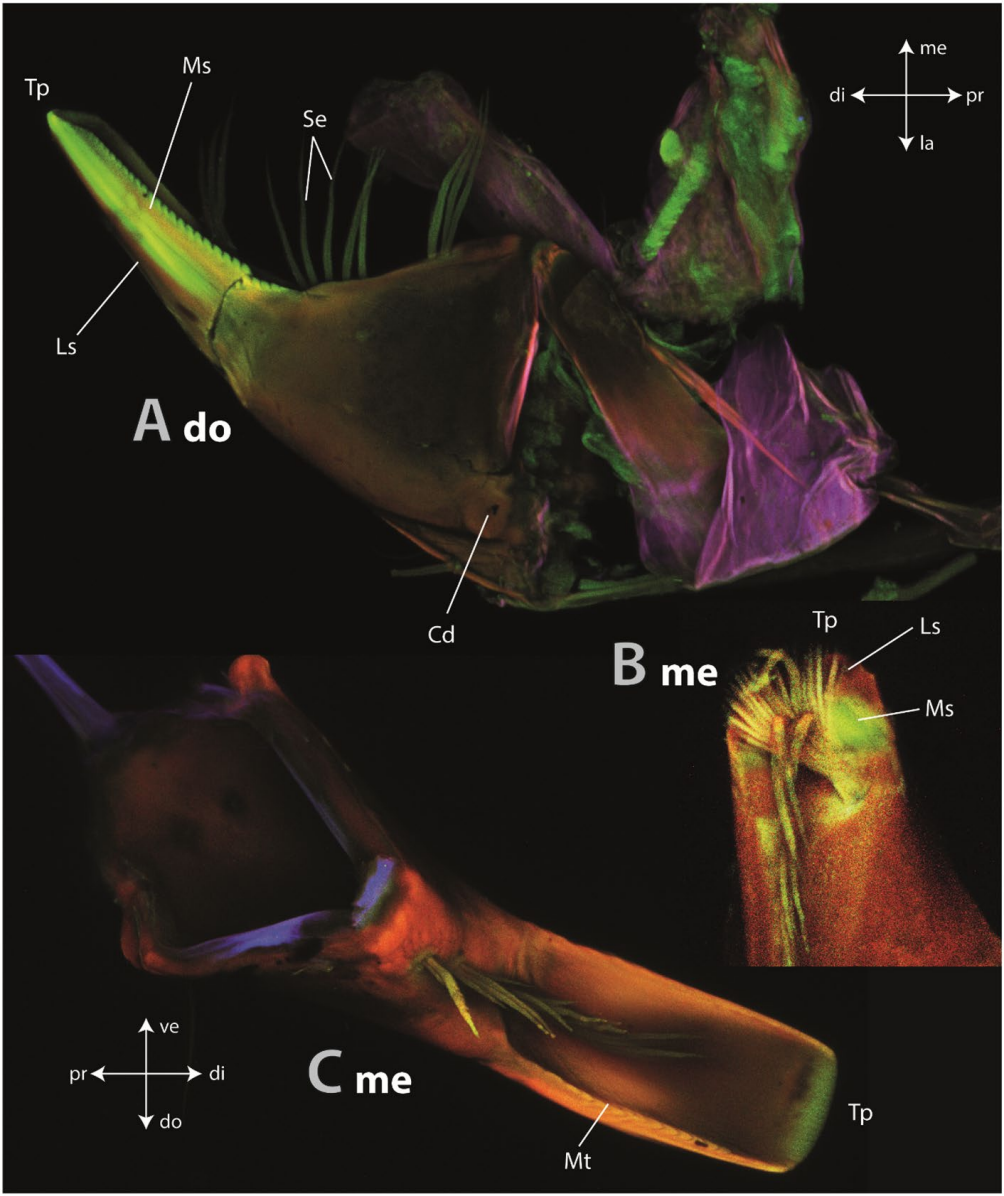
CLSM images of three different mandibles. All images were taken separately, thus the autofluorescence is not directly comparable between the images. (**A**) Dorsal view on left mandible (same specimen as in **C**). (**B**) Medial view on the left mandible with the cut-off tip. (**C**) View from medial on the right mandible (same specimen as in **A**). *Cd* dorsal condyle, *di* distal, *do* dorsal, *Ls* lateral surface, *la* lateral, *me* medial, *Mt* mandibular tooth, *pr* proximal, *Se* setae, *Tp* tip, *Os* outer surface, *ve* ventral.

Most regions of the mandible appeared red (showed a high autofluorescence from the lasers of 555 nm and 639 nm wavelength) (Fig. 4). A gradient within this structure could be determined: the condyles were of darker reddish colour, which became lighter towards the tip. The tip appeared green (showed a strong autofluorescence signal from the laser of 488 nm wavelength) (Fig. 4A,C). In dorsal view, the medial surface of the mandible was green in contrast to the lateral surface, which emitted a red signal (Fig. 4A). In the mandible with the cut-off tip, we found that the lateral surface emitted a red signal and the medial surface a green one (Fig. 4B).

The tissues surrounding the mandibles (i.e., muscles, etc.) appeared blue (showed a strong autofluorescence signal from the laser of 405 nm) to green (showed a strong autofluorescence signal from the laser of 488 nm wavelength) (Fig. 4A,C) or, when they covered part of the mandibles, were of purple colour as result from blue (tissue) and red colour (mandible) in overlay (Fig. 4A).

### Elemental analysis by EDX

**Figure 5:**
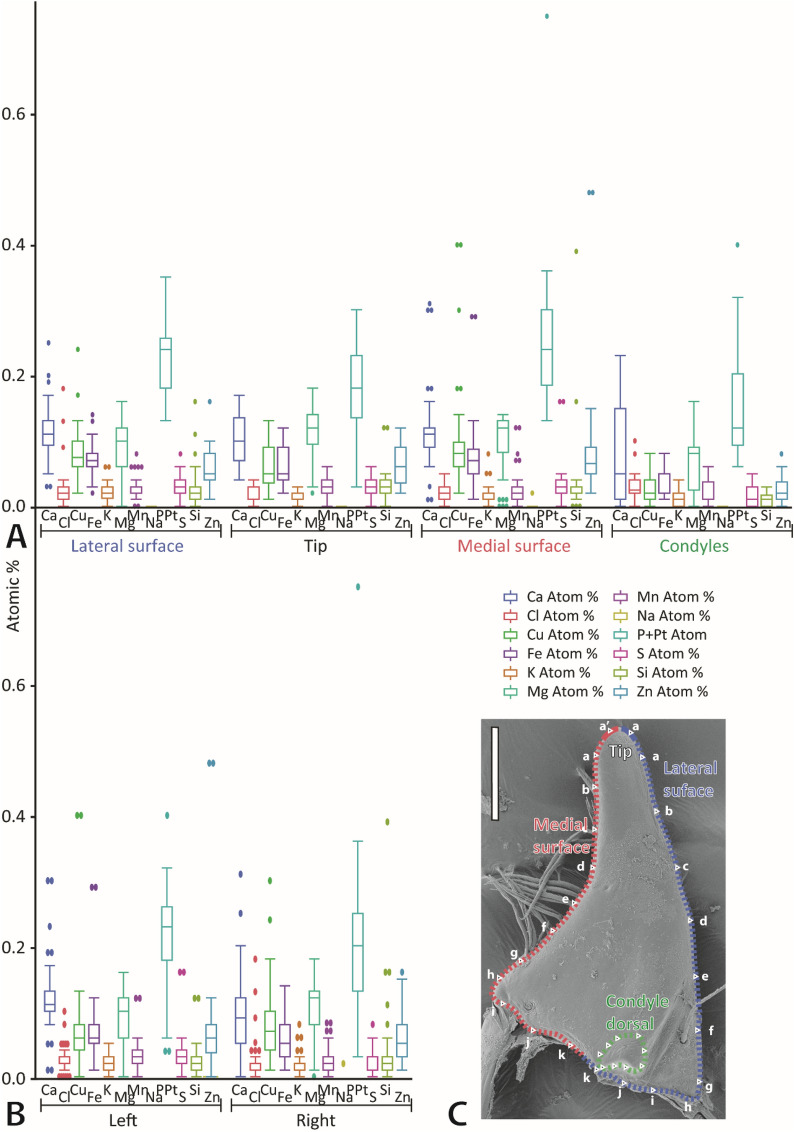
Results from EDX analysis, given in atomic %, based on the testing of two mandibles. (**A**) Elemental content of the tip, the lateral and medial surfaces and the condyles. (**B**) Results, summarized from all regions, sorted to the body side (left and right mandible are from two different specimens. (**C**) Dorsal view on one right mandible. Letters represent the localities tested (see Fig. 6 for EDX results sorted to each locality). Please note that the EDX analyses were performed on embedded and polished samples (sections of mandibles), this SEM image just illustrates the localities of the studied regions. Scale bar: 80 µm.

**Figure 6:**
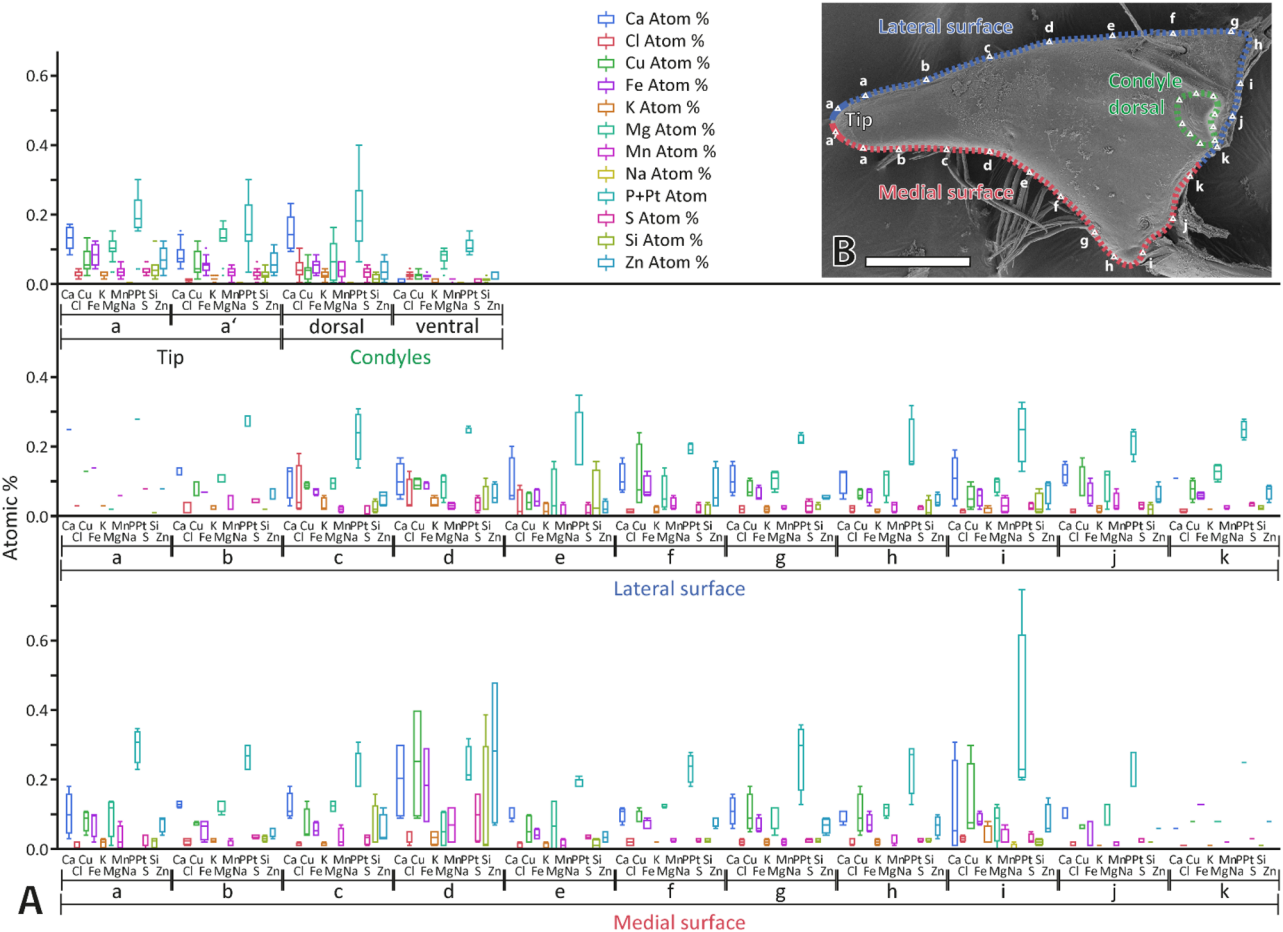
(**A**) Results from EDX analysis, given in atomic %, based on the testing of two mandibles. The results are sorted to the individual localities tested (see SEM image for the localities). (**B**) Dorsal view on one right mandible. Letters represent the localities tested. Please note that the EDX analyses were performed on embedded and polished samples (sections of mandibles). The SEM image illustrates the localities of the studied regions. Scale bar: 80 µm.

Based on the analysis of 141 point measurements, we determined Ca, Cl, Cu, Fe, K, Mg, Mn, P + Pt, S, Si, and Zn in the mandibles. All these elements were present in small proportions (< 0.30 atomic %) (see Figs. 5 and 6 and Supplementary Table 1). P + Pt (mean and standard deviation in atomic %: 0.21 ± 0.09) was detected with highest proportions, followed by Ca (0.11 ± 0.06), Mg (0.10 ± 0.05), Cu (0.07 ± 0.06), Fe (0.06 ± 0.04), Zn (0.06 ± 0.01), Si (0.03 ± 0.04), S (0.03 ± 0.02), Mn (0.03 ± 0.02), Cl (0.02 ± 0.02), and finally K (0.02 ± 0.01) with lowest proportions. Some differences between the tested regions could be determined, but we could not find a general pattern (see Supplementary Table 2 for results from pairwise comparison). When the whole content of the mandibles was compared between the two tested specimens, we could detect some differences for the proportions of Ca, Cl, Cu, Fe, K and S, but, in general, differences in proportions were rather small (see Supplementary Table 3 for means, SD and p-values). When the elements were sorted to the region and then compared between the two tested specimens, differences between the individuals were not significant (see Supplementary Table 4 for p-values). When the proportions were sorted to the tested localities, we could not detect a general pattern in the distribution as well (see Fig. 6 and Supplementary Table 5 for means and SDs).

### Mechanical properties

The Young’s modulus (*E*) describes the stiffness of a solid material and describes the relationship between axial strain and tensile stress. The hardness (*H*) is the measure of the resistance to local plastic deformation induced by indentation.

**Figure 7:**
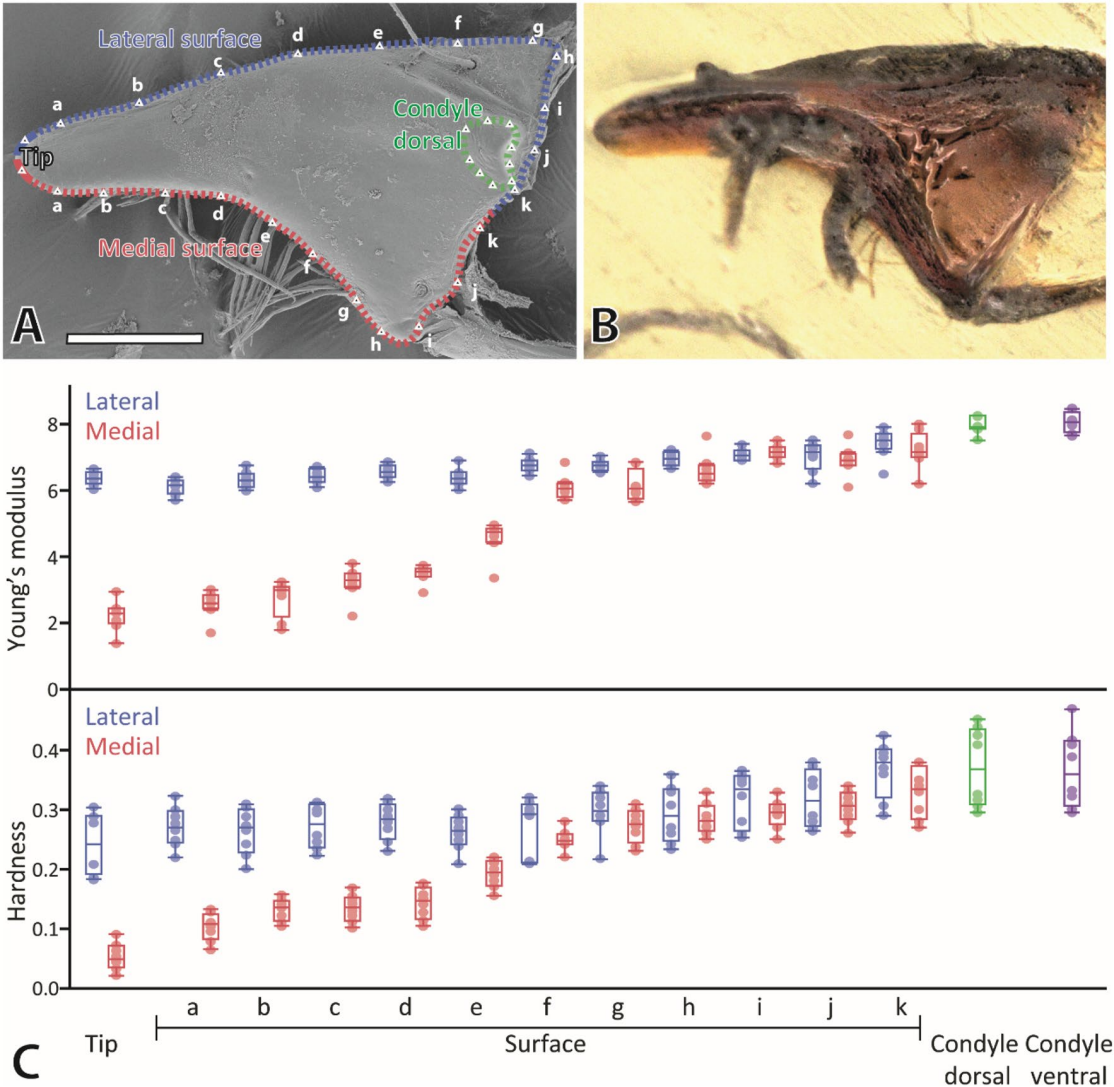
(**A**) SEM image shows the localities tested with nanoindentation. Please note that the nanoindentation was performed on embedded and polished samples (sections of mandibles); this SEM should rather illustrate the localities of the studied regions. (**B**) Light microscopy image of an embedded mandible. (**C**) Results from nanoindentation: hardness (*H*) and Young’s modulus (*E*), both given in GPa, for the tested regions on the mandible tip, medial and lateral surfaces, and both condyles. These results are based on the testing of eight mandibles. Scale bar: 80 µm.

In the mandibles, the condyles were the stiffest and hardest regions (dorsal condyle: *E* mean ± SD = 7.97 ± 0.25, *H* = 0.37 ± 0.06 GPa; ventral condyle: *E* = 8.06 ± 0.31 GPa, *H* = 0.37 ± 0.07 GPa), followed by the lateral surface of the tip (*E* = 6.35 ± 0.21 GPa; *H* = 0.24 ± 0.05 GPa), the lateral mandible surface (*E* means ranged from 6.07 to 7.41 GPa and *H* from 0.27 to 0.37 GPa), the medial mandible surface (*E* means ranged from 2.54 to 7.21 GPa; *H* from 0.10 to 0.33 GPa) and finally the medial surface of the tip as the softest and most flexible region (*E* = 2.20 ± 0.46 GPa; *H* = 0.05 ± 0.02 GPa) (see Fig. 7 and Supplementary Table 6). For the medial side of the mandibles, we determined mechanical property gradients with the medial locality (tip) as the softest and most flexible one. Towards proximal, values of *E* and *H* increased (see Fig. 7 and Supplementary Table 6 for all values). Pairwise comparison by Wilcoxon method revealed significant differences between the different regions (see Supplementary Tables 7 and 8 for p-values). For the lateral surface of the mandibles we, however, found a different pattern: values of *E* and *H* were rather similar as revealed by Wilcoxon method pairwise comparison (see Supplementary Tables 7 and 8 for p-values).

Between the individuals, *H* and *E* values, sorted to the distinct regions, were not different (see Supplementary Table 4 for p-values). Between left and right mandible, no differences with regard to *E* and *H* could be determined (see Supplementary Table 9 for p-values).

### Relationship between parameters

We determined that the following parameters were very strongly correlated (see Supplementary Table 10 for correlation coefficients): *E* and *H* (r = 0.90), Fe and Cu (r = 0.85), Zn and Cu (r = 0.90), Zn and Fe (r = 0.87). We detected strong correlations between Ca and Cu (r = 0.66), Fe and Ca (r = 0.67), K and Cl (r = 0.70), Cu and K (r = 0.61), Mn and Ca (r = 0.74), Cu and Mn (r = 0.76), Mn and Fe (r = 0.78), Ca and S (r = 0.76), Cu and S (r = 0.69), S and Fe (r = 0.79), S and Mn (r = 0.67), Zn and Ca (r = 0.60), Zn and Mn (r = 0.77), Zn and S (r = 0.78). In all other cases, correlations were either very weak, weak, or medium. No correlations between the mechanical properties and the elemental compositions were detected (see Supplementary Table 10 for correlations coefficients).

The mechanical properties were reflected by the emitted autofluorescence signal. All structures, that were harder and stiffer (e.g., the condyles) showed a strong red signal in CLSM, whereas the structures, that were softer and more flexible (e.g., the medial cuticle of the mandible tip) appeared green in CLSM.

## Discussion

Insects represent the most species-rich and diverse animal group (see^51^), are abundant in nearly all habitats and provide essential ecological functions by their actions (for review, see^52^). One reason for this diversity can be found, among other factors, in the strong variety of life history traits, which includes the foraging on different food sources or from different surfaces. Since mouthparts are interfaces between the organisms and their preferred food, research on them contributes to our understanding of insect evolution, behaviour and ecology (for review, see^53^). Insect mouthpart morphologies were intensively investigated in the past, giving important insights into functional principles and ecology (for reviews, see^52,53^). With regard to the mechanical properties (e.g., hardness and Young’s modulus), which determine the functionality of mouthparts, there are large gaps in knowledge, as only few studies address them and their origins (i.e., the degree of tanning or the inorganic content) (see^24–26,32,41^).

When investigating the adaptations in mouthparts to the food, Trichoptera larvae can be regarded as a good model system, since the larvae are ecologically highly diverse and show adaptations to grazing, shredding, filtering, or predatory behaviour (see^42,54–59^). We here investigated the mechanical properties of just one species, but, however, hope, that mechanical property tests on more species with distinct ecological niches will follow.

### Mechanical properties

In insects, the mechanical properties can show a large range of values across units from KPa to GPa (see review^25,60^). This depends on the tested region, as the cuticle is a heterogeneous composite material, and on the water content^8,61–63^. Structures that are less prone to abrasion, e.g., legs, eyes, elytra or wings^64–70^ are usually softer and more flexible, whereas e.g., mouthparts, joints or claws are usually harder and stiffer (see^29,71–76^).

**Figure 8:**
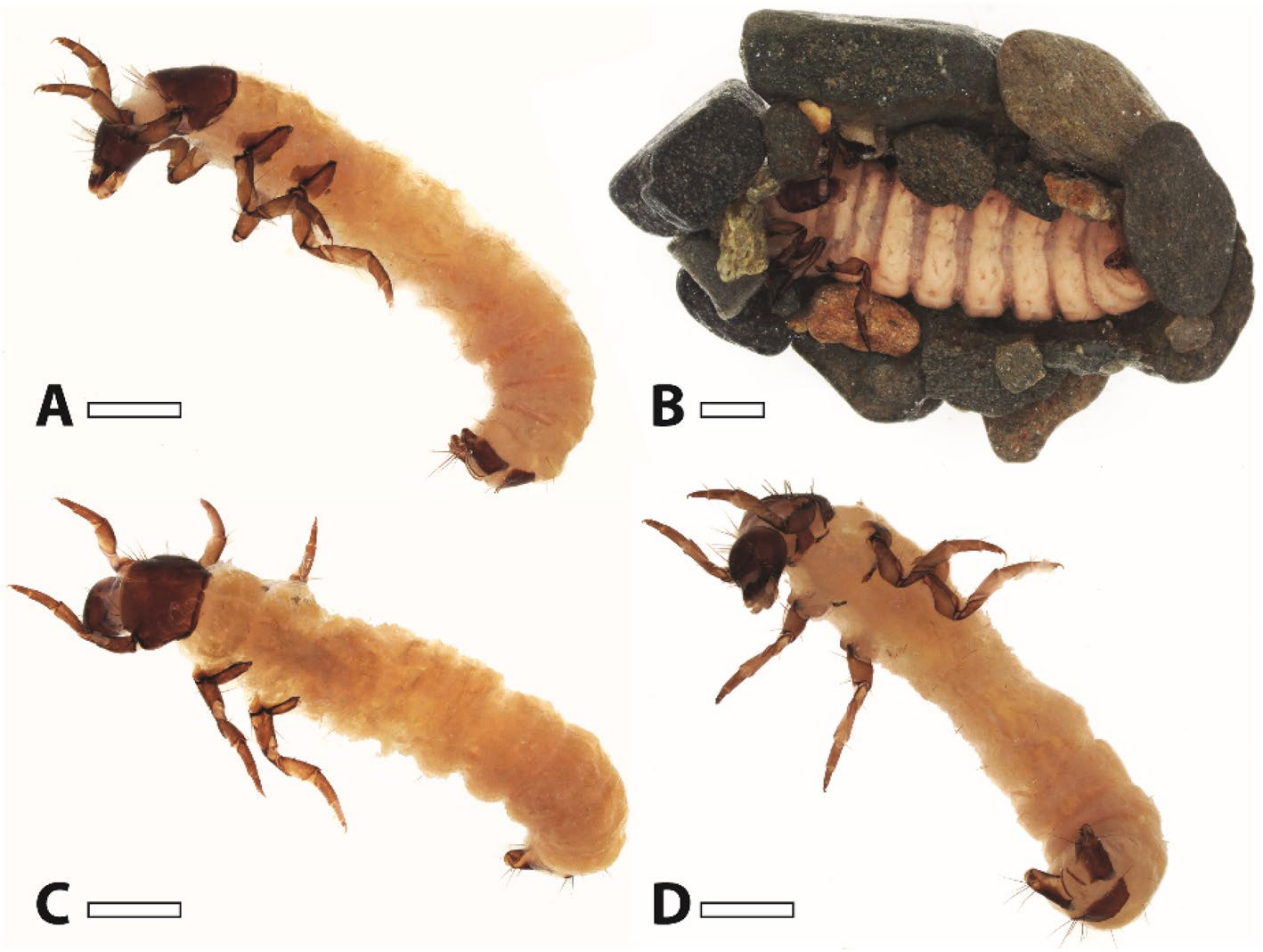
Light microscopy images of larval *Glossosoma boltoni*. (**A**) Lateral view. (**B**) Larva with the protective case. (**C**) Dorsal view. (**D**) Ventral view. Scale bars: 1 mm.

In mandibles, *E* values from ~ 6 to ~ 11 GPa and *H* values from 0.4 to 1.2 GPa were documented in termites^73^, *E* values from ~ 7 to ~ 11 GPa and *H* values from ~ 0.7 to ~ 1.0 GPa in dragonfly nymphs^76^, *E* values from ~ 7 to ~ 11 GPa and *H* values from ~ 0.3 to ~ 1.0 GPa in beetle larvae^77^, and *E* values from ~ 3.5 to ~ 20.9 GPa and *H* values from ~ 0.2 to ~ 2.0 GPa in antlion larvae^41^. The maximum values of *E* and *H* of *Glossosoma boltoni* (see Fig. 8 for habitus) are in general within the range of ant, dragonfly nymph and beetle larvae mouthparts. However, in *G. boltoni* the mandible tip, which interacts with the ingesta, was softer and more flexible inside and harder and stiffer outside, which was not detected for the other insect mandibles tested by nanoindentation; they show decreasing mechanical property values from the interacting mouthpart regions towards the mouthpart basis.

### Origin of mechanical property gradients

The heterogeneity in mechanical properties of insect cuticle (see review^32^) can come from the degree of sclerotization by quinone reactions^24,78^, the chitin microstructure, the abundance of the mineral content (abundant in small proportions), proteins, transition metals and halogens (^28,29^; for review on mechanical property gradients and their various origins, see^31^).

The insect cuticle usually lacks higher proportions of inorganic contents, in contrast to other arthropods. However, transition metals (like Cu, Fe, Mn, Zn) with colocalized halogens (Cl) are abundant in small proportions and probably act as cross-linkers^37,39,71,79–84^. Additionally, alkaline earth metals (Ca, Mg), potentially as part of biomineralization, can be present in small proportions^27,38–41^. P, Si, Si, Cl, K or Na, can also be detected in such localities^27,40,74,85–95^. As especially Zn, Mn, Ca and Mg seem to relate directly to an increase in hardness and thus wear resistance in insects^28,29,41,71,73,74,77,78,92,96–98^, such components were found in structures that are prone to structural failure or wear. Examples for this are claws^74^, ovipositors^40,94,99^ and mouthparts^28,29,71,73,82,95,100–106^. In *Glossosoma boltoni*, we did not detect high proportions of transition and earth alkaline metals (see Figs. 5 and 6 and Supplementary Table 1). Additionally, no correlations between elemental content and mechanical property values could be determined (see Supplementary Table 10 for correlation coefficients). Potentially, relationships could be determined when increasing the sample size or when using younger samples.

The degree of sclerotisation and its relationship with the mechanical properties were well investigated in cuticle structures. Autofluorescence signals, received after laser excitation via CLSM according to the protocol of^49^, are commonly studied to identify cuticle regions with certain dominating material compositions. Sclerotized and stiff cuticle is related to the signal received after excitation with lasers of 555 nm and 639 nm wavelength (here, the red colour was assigned to the signal). Less sclerotized chitin-rich cuticle, which is flexible and relatively tough, emits signals from the laser of 488 nm wavelength (here, the green colour was assigned to the signal). Regions with high proportions of resilin, an elastic and flexible protein (see^107^; for review see^108^), or other proteins^109,110^ emit a strong signal after excitement with the laser of 405 nm wavelength (here, the blue colour was assigned to the signal). Regions of mixed material compositions can appear brown, yellow, or pink from overlay of different signals. CLSM protocols, mostly following^49^, were previously applied to chitinous structures, such as wings^111^, foot attachment devices^112^, antennae^113^, genitals^114^, and also mouthparts (^115–120^; for Trichoptera, see^121^).

A relationship between the autofluorescence signals and the material properties (see^122^) was previously cross-validated via AFM-nanoindentation for leg attachment devices in lady bird beetles^112^, antlion larvae mandibles^41^ and for chitinous radular teeth of gastropod molluscs^50^. The same relationship is also detected here, as we determined that blue to green areas are softer and more flexible (e.g., the medial surface of the mandible tip) than regions of red colour (e.g., the bases of the mandible or the lateral surface of the mandible tip), which suggests that the mechanical property gradients in *Glossosoma boltoni* have their origin in the degree of tanning. This relationship was previously also suggested by^11^ for the mandibles of this species.

### Interaction with hard substrate

Interaction with hard substrate is challenging due to the risk of structural failure or wear. The chitinous molluscan teeth are among the best studied structures that interact directly with hard and abrasive ingesta as e.g. stones. Here, the teeth can be heavily mineralized with iron oxides and/or silica (Polyplacophora and Patellogastropoda), which are located between the chitin fibres and increase the hardness and elasticity dramatically (for reviews, see^13,14,123^). However, in some gastropod taxa (e.g. Paludomidae) foraging from stones, teeth are not heavily mineralized^50,124,125^. Here, teeth can withstand high forces by their clever geometry and mechanical property gradients, which allows the teeth of one row to bend and to gain support from the adjacent row distributing the stress^8,63^. Additionally, the tooth tips show a high degree of cross-linking of the chitin fibres (sclerotization), which seems to reduce the degree of wear^50^. In *G. boltoni*, we did not determine high proportions of metals, but found by CLSM that the mechanical property gradients probably originate from the degree of tanning. This system seems to be analogous to the not-mineralized radular teeth.

From the wear distribution we could receive data on the regions that interact with the hard substrate during foraging. As the medial surface of the mandible tip showed sign of heavy wear (Fig. 2F), we propose that this species performs a scooping motion on the rocks to remove algae. The wear on the outer mandible surface (the lateral side; see Fig. 2A–E) could potentially come from interactions with adjacent sand particles or adjacent smaller stones. We could not identify wear on the mandible teeth (Fig. 1H), which indicates that they are not used for loosening algae from stones; they are potentially involved in cutting larger algae.

### Self-sharpening and function

Due to the abrasive particles or substrates (ingesta), wear on a cutting edge of tools or structures can affect its sharpness and hence functionality. Adaptations to prevent or reduce dulling can be found in teeth of beavers^126,127^, *Triceratops*^128^, sea urchins^3,5,7^ and molluscs^1,4,6^. Additionally, self-sharpening mechanisms were previously determined for the mandibles of the locust *Locusta migratoria* by mechanical property tests^96^ and proposed for the mandibles of beetles by documenting the geometry^77,84^. Based on the data from^96^, insect mandibles were already on focus in the development of self-sharpening cutting tools^129^.

In general, self-sharpening mechanisms, which are also applied to bionic tribological systems (see^130,131^), result from the following: the hardness is not uniform within structure. One part (e.g., the layers of enamel on the beaver incisor surface, see^127^), which is hard and wear-resistant, interacts with the food or substrate. The second part, which is rather soft, can withstand possible impacts, compressive and tensile stresses (e.g., the dentine underneath the enamel in beaver incisor, see^127^). However, this region shows a high degree of wear. Due to weaker interfaces between the harder and the softer region, cracks produced during feeding can propagate along the interfaces more easily than perpendicular to them (see^3^). This heterogeneity can be achieved by the microstructure of the organic matrix and the distribution of inorganics and results in a sharpening of the tip profile.

In *Glossosoma boltoni*, we could not test the interfacial strengthening, but determined mechanical property heterogeneities between the medial and the lateral mandible surface (cuticle). The mechanical property distribution of *G*. *boltoni* is different to the one determined in the self-sharpening mandibles of the locust *Locusta migratoria*; here the lateral surface of one mandible tip was harder than the other and the medial surface of the counter mandible tip was harder than the other^96^.

However, the medial surface of *G. boltoni* was significantly softer, more flexible and showed pronounced signs of wear, similar to the situation in beavers. In contrast, the lateral surface, which presumably interacts with the stone during feeding, was significantly harder, stiffer and showed less wear, similar to the situation in beavers as well. Potentially, when the mandible tip scratches across the stone surface to loosen algae, the soft medial cuticle abrades faster than the harder lateral cuticle, which would lead to a sharp cutting edge at the mandible tip. By SEM we found that mandible tips of *G. boltoni* appeared rather sharp in profile, similar to the situation found in some beetle mandibles that are thought to be self-sharpened as well^77,84^. Due to these indications, we suggest that a self-sharpening mechanism is present in the mandibles of these trichopteran larvae. However, this should be verified in the future, potentially in the form of in situ wear studies, similar to studies on sea urchin teeth^7^.

## Conclusion

We studied the mandibles of a trichopteran larvae foraging from stones by scanning electron microscopy, confocal laser scanning microscopy, energy dispersive X-ray spectroscopy and nanoindentation. We found regional heterogeneities in mechanical properties: the softest and most flexible region was the medial surface of the mandible tip, which showed high degrees of wear, whereas the lateral region was hard and stiff. Due to the presence of these gradients, which were found to relate to the degree of tanning, we suggest that potentially a self-sharpening mechanism is present in these mouthparts facilitating the foraging on challenging surfaces.

### Supplementary Information

Supplementary Tables.

## Data Availability

The raw data is available from the corresponding author on reasonable request, but all values can be found in the Supplementary.
