# Supplementary material for: Mandible mechanical properties and composition of the larval Glossosoma boltoni (Trichoptera, Insecta)
Source: Sci Rep. 2024 Feb 26;14:4695. doi: 10.1038/s41598-024-55211-5 (PMC10897335; doi:10.1038/s41598-024-55211-5)
Supplement: Supplementary file 1 — Supplementary Tables. [file 41598_2024_55211_MOESM1_ESM.pdf]

Mandible mechanical properties and composition of the larval *Glossosoma boltoni* (Trichoptera, Insecta)

Wencke Krings[1,2,3,4*], Patrick Below[2,3], Stanislav N. Gorb[4]

[1] Department of Cariology, Endodontology and Periodontology, Universität Leipzig, Liebigstraße 12, 04103 Leipzig, Germany
[2] Department of Electron Microscopy, Institute of Cell and Systems Biology of Animals, Universität Hamburg, Martin-Luther-King-Platz 3, 20146 Hamburg, Germany
[3] Department of Mammalogy and Palaeoanthropology, Leibniz Institute for the Analysis of Biodiversity Change, Martin-Luther-King-Platz 3, 20146 Hamburg, Germany
[4] Department of Functional Morphology and Biomechanics, Zoological Institute, Christian-Albrechts-Universität zu Kiel, Am Botanischen Garten 1-9, 24118 Kiel, Germany

*corresponding author: wencke.krings@uni-hamburg.de

Supplementary materials

Supplementary Table 1. Results from EDX analyses, mean and SD for each individual element analyzed, sorted to the regions. Abbreviations: SD, standard deviation.

Region	N	Ca mean	Ca SD	Cl mean	Cl SD	Cu mean	Cu SD	Fe mean	Fe SD	K mean	K SD	Mg mean	Mg SD
Lateral surface	40	0.11	0.04	0.02	0.03	0.07	0.04	0.06	0.02	0.02	0.01	0.09	0.04
Tip	41	0.10	0.03	0.01	0.01	0.06	0.03	0.06	0.02	0.01	0.00	0.11	0.03
Medial surface	40	0.11	0.06	0.02	0.01	0.10	0.08	0.07	0.05	0.02	0.01	0.09	0.04
Condyle	20	0.07	0.07	0.03	0.02	0.02	0.01	0.03	0.01	0.01	0.01	0.06	0.04

Region	N	Mn mean	Mean SD	P+Pt mean	P+Pt SD	S mean	S SD	Si mean	Si SD	Zn mean	Zn SD
Lateral surface	40	0.02	0.01	0.22	0.05	0.02	0.01	0.02	0.03	0.05	0.02
Tip	41	0.02	0.01	0.18	0.06	0.03	0.01	0.03	0.02	0.06	0.03
Medial surface	40	0.02	0.02	0.25	0.09	0.03	0.03	0.03	0.06	0.08	0.09
Condyle	20	0.02	0.01	0.15	0.08	0.01	0.01	0.01	0.00	0.02	0.02

Supplementary Table 2. Results from Kruskal-Wallis-test and p-values from pairwise comparison by Wilcoxon method for each individual element, sorted according to the region. p-values: $p < 0.001$ = highly significant differences; $0.05 < p < 0.10$ = significant differences; $p > 0.10$ = no significant differences.

Element	Region 1	Region 2	Results from Kruskal-Wallis-test			p-value
			Chi²	DF	p-value	
Ca	Medial surface	Tip	5.3466	3	0.1481	0.5759
Ca	Medial surface	Lateral surface				0.7825
Ca	Tip	Lateral surface				0.5192
Ca	Condyle	Tip				0.1091
Ca	Condyle	Medial surface				0.0435*
Ca	Condyle	Lateral surface				0.0317*
Cl	Condyle	Tip	9.2216	3	0.0265*	0.0082*
Cl	Medial surface	Tip				0.0993
Cl	Condyle	Medial surface				0.1169
Cl	Condyle	Lateral surface				0.3246
Cl	Medial surface	Lateral surface				0.5131
Cl	Tip	Lateral surface				0.0239*
Cu	Medial surface	Tip	40.7799	3	<.0001*	0.0037*
Cu	Medial surface	Lateral surface				0.2696
Cu	Tip	Lateral surface				0.0256*
Cu	Condyle	Tip				<.0001*
Cu	Condyle	Lateral surface				<.0001*
Cu	Condyle	Medial surface				<.0001*
Fe	Medial surface	Tip	25.7666	3	<.0001*	0.2663
Fe	Medial surface	Lateral surface				0.7519
Fe	Tip	Lateral surface				0.1939

Fe	Condyle	Tip				<.0001*
Fe	Condyle	Medial surface				<.0001*
Fe	Condyle	Lateral surface				<.0001*
K	Medial surface	Tip	11.7691	3	0.0082*	0.3035
K	Condyle	Tip				0.1719
K	Medial surface	Lateral surface				0.1104
K	Condyle	Medial surface				0.0522
K	Tip	Lateral surface				0.0104*
K	Condyle	Lateral surface				0.0045*
Mg	Tip	Lateral surface	16.7947	3	0.0008*	0.0166*
Mg	Medial surface	Lateral surface				0.2066
Mg	Medial surface	Tip				0.2162
Mg	Condyle	Lateral surface				0.0220*
Mg	Condyle	Medial surface				0.0074*
Mg	Condyle	Tip				0.0001*
Mn	Tip	Lateral surface	8.5103	3	0.0366*	0.8612
Mn	Condyle	Medial surface				0.1477
Mn	Medial surface	Lateral surface				0.1138
Mn	Medial surface	Tip				0.0853
Mn	Condyle	Lateral surface				0.0286*
Mn	Condyle	Tip				0.0181*
P+Pt	Medial surface	Tip	32.0846	3	<.0001*	<.0001*
P+Pt	Medial surface	Lateral surface				0.2204
P+Pt	Condyle	Tip				0.0218*
P+Pt	Tip	Lateral surface				0.0018*
P+Pt	Condyle	Lateral surface				<.0001*
P+Pt	Condyle	Medial surface				<.0001*
S	Medial surface	Lateral surface	14.8324	3	0.0020*	0.5448
S	Tip	Lateral surface				0.6227
S	Medial surface	Tip				0.9333
S	Condyle	Lateral surface				0.0026*
S	Condyle	Tip				0.0007*
S	Condyle	Medial surface				0.0004*
Si	Tip	Lateral surface	20.4182	3	<.0001*	0.1004
Si	Medial surface	Lateral surface				0.3388
Si	Medial surface	Tip				0.2018
Si	Condyle	Lateral surface				0.0038*
Si	Condyle	Medial surface				0.0001*
Si	Condyle	Tip				<.0001*
Zn	Medial surface	Lateral surface	27.4035	3	<.0001*	0.1722
Zn	Medial surface	Tip				0.5150
Zn	Tip	Lateral surface				0.5980
Zn	Condyle	Lateral surface				<.0001*
Zn	Condyle	Tip				<.0001*
Zn	Condyle	Medial surface				<.0001*

Supplementary Table 3. Results from EDX analyses. Means and SDs for each individual element analyzed, sorted according to the left (from specimen A) and right mandible (from specimen B). Results from pairwise comparison by Wilcoxon method. p-values: p < 0.001 = highly significant differences; 0.05 < p < 0.10 = significant differences; p > 0.10 = no significant differences. Abbreviations: SD, standard deviation.

Left or right mandible	N	Ca mean	Ca SD	Chi^2	DF	p-value	Cl mean	Cl SD	Chi^2	DF	p-value	Cu mean	Cu SD	Chi^2	DF	p-value
Left	70	0.12	0.04	14.5450	1	<.0001*	0.03	0.01	18.2678	1	<.0001*	0.07	0.06	1.7067	1	<.0001*
Right	71	0.09	0.06				0.02	0.02				0.08	0.05			
**Left or right mandible**	**N**	**Fe mean**	**Fe SD**	**Chi^2**	**DF**	**p-value**	**K mean**	**K SD**	**Chi^2**	**DF**	**p-value**	**Mg mean**	**Mg SD**	**Chi^2**	**DF**	**p-value**
Left	70	0.07	0.04	2.6235	1	<.0001*	0.02	0.01	6.8781	1	0.0087*	0.09	0.04	2.3922	1	0.1219
Right	71	0.06	0.03				0.02	0.01				0.10	0.04			
**Left or right mandible**		**Mn mean**	**Mn SD**	**Chi^2**	**DF**	**p-value**	**P+Pt mean**	**P+Pt SD**	**Chi^2**	**DF**	**p-value**	**S mean**	**S SD**	**Chi^2**	**DF**	**p-value**
Left	70	0.03	0.02	1.4806	1	0.2237	0.22	0.07	3.0508	1	0.0807	0.03	0.03	9.0596	1	0.0026*
Right	71	0.03	0.01				0.21	0.10				0.03	0.02			
**Left or right mandible**		**Si mean**	**Si SD**	**Chi^2**	**DF**	**p-value**	**Zn mean**	**Zn SD**	**Chi^2**	**DF**	**p-value**					
Left	70	0.02	0.02	0.0690	1	0.7928	0.07	0.07	0.1632	1	0.6862					
Right	71	0.03	0.05				0.06	0.03								

Supplementary Table 4. P-values from pairwise comparison by Wilcoxon method between the tested specimens for the results from EDX and nanoindentation, sorted to the region. p-values: $p < 0.001$ = highly significant differences; $0.05 < p < 0.10$ = significant differences; $p > 0.10$ = no significant differences.

Parameter	Structure	Specimen 1	Specimen 2	p-value
Ca	Condyle, dorsal	A	B	1.0000
Ca	Condyle, ventral	A	B	1.0000
Ca	Mandible tip, lateral surface	A	B	1.0000
Ca	Mandible tip, medial surface	A	B	1.0000
Ca	Mandible, lateral surface	A	B	0.6985
Ca	Mandible, medial surface	A	B	0.6985
Cl	Condyle, dorsal	A	B	1.0000
Cl	Condyle, ventral	A	B	1.0000
Cl	Mandible tip, lateral surface	A	B	0.6985
Cl	Mandible tip, medial surface	A	B	1.0000
Cl	Mandible, lateral surface	A	B	0.6985
Cl	Mandible, medial surface	A	B	0.6985
Cu	Condyle, dorsal	A	B	1.0000
Cu	Condyle, ventral	A	B	0.6985
Cu	Mandible tip, lateral surface	A	B	1.0000
Cu	Mandible tip, medial surface	A	B	1.0000
Cu	Mandible, lateral surface	A	B	1.0000
Cu	Mandible, medial surface	A	B	1.0000
Fe	Condyle, dorsal	A	B	1.0000
Fe	Condyle, ventral	A	B	1.0000
Fe	Mandible tip, lateral surface	A	B	1.0000
Fe	Mandible tip, medial surface	A	B	0.6985
Fe	Mandible, lateral surface	A	B	0.8217
Fe	Mandible, medial surface	A	B	0.8217
K	Condyle, dorsal	A	B	0.8217
K	Condyle, ventral	A	B	0.8217
K	Mandible tip, lateral surface	A	B	0.0986
K	Mandible tip, medial surface	A	B	0.8217
K	Mandible, lateral surface	A	B	0.0986
K	Mandible, medial surface	A	B	0.0986
Mg	Condyle, dorsal	A	B	0.6171
Mg	Condyle, ventral	A	B	1.0000
Mg	Mandible tip, lateral surface	A	B	0.0986
Mg	Mandible tip, medial surface	A	B	1.0000
Mg	Mandible, lateral surface	A	B	0.0986
Mg	Mandible, medial surface	A	B	1.0000
Mn	Condyle, dorsal	A	B	1.0000
Mn	Condyle, ventral	A	B	1.0000
Mn	Mandible tip, lateral surface	A	B	0.8217
Mn	Mandible tip, medial surface	A	B	0.4142
Mn	Mandible, lateral surface	A	B	0.4142
Mn	Mandible, medial surface	A	B	0.4142
P+Pt	Condyle, dorsal	A	B	0.6985
P+Pt	Condyle, ventral	A	B	1.0000
P+Pt	Mandible tip, lateral surface	A	B	1.0000
P+Pt	Mandible tip, medial surface	A	B	1.0000
P+Pt	Mandible, lateral surface	A	B	0.6985
P+Pt	Mandible, medial surface	A	B	0.6985
S	Condyle, dorsal	A	B	0.6985
S	Condyle, ventral	A	B	1.0000
S	Mandible tip, lateral surface	A	B	0.6985
S	Mandible tip, medial surface	A	B	1.0000
S	Mandible, lateral surface	A	B	1.0000
S	Mandible, medial surface	A	B	1.0000
Si	Condyle, dorsal	A	B	0.6985
Si	Condyle, ventral	A	B	1.0000
Si	Mandible tip, lateral surface	A	B	1.0000
Si	Mandible tip, medial surface	A	B	1.0000
Si	Mandible, lateral surface	A	B	0.6171
Si	Mandible, medial surface	A	B	1.0000
Zn	Condyle, dorsal	A	B	1.0000

Zn	Condyle, ventral	A	B	1.0000
Zn	Mandible tip, lateral surface	A	B	0.8217
Zn	Mandible tip, medial surface	A	B	0.0986
Zn	Mandible, lateral surface	A	B	1.0000
Zn	Mandible, medial surface	A	B	1.0000
E	Condyle, dorsal	B	A	0.2453
E	Condyle, dorsal	C	A	0.2453
E	Condyle, dorsal	C	B	0.6985
E	Condyle, dorsal	D	A	0.2453
E	Condyle, dorsal	D	B	0.6985
E	Condyle, dorsal	D	C	0.6985
E	Condyle, dorsal	E	A	0.6985
E	Condyle, dorsal	E	B	1.0000
E	Condyle, dorsal	E	C	1.0000
E	Condyle, dorsal	E	D	1.0000
E	Condyle, ventral	B	A	0.6985
E	Condyle, ventral	C	A	0.2453
E	Condyle, ventral	C	B	0.2453
E	Condyle, ventral	D	A	0.2453
E	Condyle, ventral	D	B	0.2453
E	Condyle, ventral	D	C	0.6985
E	Condyle, ventral	E	A	0.2453
E	Condyle, ventral	E	B	0.6985
E	Condyle, ventral	E	C	0.2453
E	Condyle, ventral	E	D	1.0000
E	Mandible tip, lateral surface	B	A	0.2453
E	Mandible tip, lateral surface	C	A	0.2453
E	Mandible tip, lateral surface	C	B	1.0000
E	Mandible tip, lateral surface	D	A	0.2453
E	Mandible tip, lateral surface	D	B	0.6985
E	Mandible tip, lateral surface	D	C	0.6985
E	Mandible tip, lateral surface	E	A	0.6985
E	Mandible tip, lateral surface	E	B	0.6985
E	Mandible tip, lateral surface	E	C	0.6985
E	Mandible tip, lateral surface	E	D	1.0000
E	Mandible tip, medial surface	B	A	0.2453
E	Mandible tip, medial surface	C	A	0.2453
E	Mandible tip, medial surface	C	B	1.0000
E	Mandible tip, medial surface	D	A	0.2453
E	Mandible tip, medial surface	D	B	1.0000
E	Mandible tip, medial surface	D	C	0.6985
E	Mandible tip, medial surface	E	A	1.0000
E	Mandible tip, medial surface	E	B	0.6985
E	Mandible tip, medial surface	E	C	0.6985
E	Mandible tip, medial surface	E	D	0.6985
E	Mandible, lateral surface	B	A	0.6472
E	Mandible, lateral surface	C	A	0.1131
E	Mandible, lateral surface	C	B	0.2751
E	Mandible, lateral surface	D	A	0.1361
E	Mandible, lateral surface	D	B	0.2453
E	Mandible, lateral surface	D	C	0.9159
E	Mandible, lateral surface	E	A	1.0000
E	Mandible, lateral surface	E	B	0.9159
E	Mandible, lateral surface	E	C	0.9159
E	Mandible, lateral surface	E	D	0.9159
E	Mandible, medial surface	B	A	0.4740
E	Mandible, medial surface	C	A	0.5812
E	Mandible, medial surface	C	B	0.7692
E	Mandible, medial surface	D	A	0.5812
E	Mandible, medial surface	D	B	0.8603
E	Mandible, medial surface	D	C	0.9159
E	Mandible, medial surface	E	A	0.8603
E	Mandible, medial surface	E	B	0.9159
E	Mandible, medial surface	E	C	0.9159
E	Mandible, medial surface	E	D	0.8603
H	Condyle, dorsal	B	A	0.6985
H	Condyle, dorsal	C	A	0.6985
H	Condyle, dorsal	C	B	0.6985

H	Condyle, dorsal	D	A	0.6985
H	Condyle, dorsal	D	B	0.6985
H	Condyle, dorsal	D	C	0.6985
H	Condyle, dorsal	E	A	1.0000
H	Condyle, dorsal	E	B	1.0000
H	Condyle, dorsal	E	C	1.0000
H	Condyle, dorsal	E	D	1.0000
H	Condyle, ventral	B	A	1.0000
H	Condyle, ventral	C	A	0.6985
H	Condyle, ventral	C	B	0.6985
H	Condyle, ventral	D	A	1.0000
H	Condyle, ventral	D	B	1.0000
H	Condyle, ventral	D	C	1.0000
H	Condyle, ventral	E	A	0.6985
H	Condyle, ventral	E	B	0.6985
H	Condyle, ventral	E	C	0.6985
H	Condyle, ventral	E	D	1.0000
H	Mandible tip, lateral surface	B	A	0.2453
H	Mandible tip, lateral surface	C	A	0.2453
H	Mandible tip, lateral surface	C	B	0.6985
H	Mandible tip, lateral surface	D	A	0.2453
H	Mandible tip, lateral surface	D	B	1.0000
H	Mandible tip, lateral surface	D	C	0.6985
H	Mandible tip, lateral surface	E	A	0.6985
H	Mandible tip, lateral surface	E	B	1.0000
H	Mandible tip, lateral surface	E	C	1.0000
H	Mandible tip, lateral surface	E	D	1.0000
H	Mandible tip, medial surface	B	A	0.6985
H	Mandible tip, medial surface	C	A	0.2453
H	Mandible tip, medial surface	C	B	0.2453
H	Mandible tip, medial surface	D	A	0.2453
H	Mandible tip, medial surface	D	B	0.2453
H	Mandible tip, medial surface	D	C	1.0000
H	Mandible tip, medial surface	E	A	1.0000
H	Mandible tip, medial surface	E	B	1.0000
H	Mandible tip, medial surface	E	C	1.0000
H	Mandible tip, medial surface	E	D	1.0000
H	Mandible, lateral surface	B	A	0.5416
H	Mandible, lateral surface	C	A	0.4247
H	Mandible, lateral surface	C	B	0.4960
H	Mandible, lateral surface	D	A	0.1885
H	Mandible, lateral surface	D	B	0.3072
H	Mandible, lateral surface	D	C	0.6812
H	Mandible, lateral surface	E	A	0.9159
H	Mandible, lateral surface	E	B	0.9159
H	Mandible, lateral surface	E	C	0.9159
H	Mandible, lateral surface	E	D	0.9159
H	Mandible, medial surface	B	A	0.8694
H	Mandible, medial surface	C	A	0.8880
H	Mandible, medial surface	C	B	0.8787
H	Mandible, medial surface	D	A	0.9719
H	Mandible, medial surface	D	B	0.8787
H	Mandible, medial surface	D	C	0.9813
H	Mandible, medial surface	E	A	0.9159
H	Mandible, medial surface	E	B	0.9159
H	Mandible, medial surface	E	C	0.9159
H	Mandible, medial surface	E	D	0.9159

Supplementary Table 5. Results from EDX analyses, mean and SD for each individual element analyzed, sorted to the locality. Abbreviations: SD, standard deviation.

Region	Locality	N	Ca mean	Ca SD	Cl mean	Cl SD	Cu mean	Cu SD	Fe mean	Fe SD	K mean	K SD	Mg mean	Mg SD
Lateral surface	a	1	0.25	0.00	0.03	0.00	0.13	0.00	0.14	0.00	0.03	0.00	0.02	0.00
Lateral surface	b	3	0.12	0.01	0.03	0.01	0.07	0.02	0.07	0.00	0.02	0.00	0.10	0.01
Lateral surface	c	4	0.10	0.05	0.07	0.07	0.09	0.00	0.07	0.00	0.03	0.01	0.09	0.01
Lateral surface	d	4	0.10	0.04	0.05	0.04	0.09	0.01	0.08	0.01	0.04	0.01	0.09	0.03
Lateral surface	e	4	0.09	0.07	0.03	0.04	0.04	0.02	0.05	0.02	0.01	0.02	0.05	0.07
Lateral surface	f	4	0.11	0.04	0.01	0.00	0.10	0.09	0.08	0.03	0.01	0.00	0.06	0.05
Lateral surface	g	4	0.10	0.04	0.02	0.01	0.07	0.02	0.06	0.01	0.02	0.00	0.10	0.02
Lateral surface	h	4	0.10	0.03	0.02	0.00	0.06	0.01	0.06	0.02	0.01	0.00	0.10	0.04
Lateral surface	l	4	0.11	0.06	0.01	0.00	0.05	0.03	0.05	0.02	0.01	0.01	0.09	0.02
Lateral surface	j	4	0.12	0.02	0.01	0.00	0.08	0.05	0.06	0.03	0.02	0.00	0.09	0.05
Lateral surface	k	4	0.11	0.00	0.01	0.00	0.07	0.02	0.06	0.00	0.02	0.00	0.12	0.02
Tip, Medial surface	a	20	0.13	0.02	0.02	0.00	0.06	0.03	0.07	0.02	0.02	0.00	0.09	0.03
Tip, Lateral surface	a‘	21	0.08	0.02	0.00	0.00	0.05	0.03	0.04	0.02	0.01	0.00	0.12	0.03
Lateral surface	a	4	0.10	0.06	0.01	0.01	0.08	0.02	0.07	0.03	0.01	0.01	0.09	0.05
Lateral surface	b	4	0.13	0.00	0.02	0.00	0.07	0.00	0.05	0.02	0.02	0.00	0.13	0.02
Lateral surface	c	4	0.12	0.03	0.01	0.00	0.06	0.04	0.05	0.02	0.01	0.00	0.12	0.01
Lateral surface	d	4	0.20	0.11	0.04	0.02	0.25	0.17	0.18	0.12	0.03	0.02	0.05	0.05
Lateral surface	e	4	0.11	0.02	0.00	0.00	0.05	0.04	0.04	0.01	0.00	0.00	0.06	0.07
Lateral surface	f	4	0.10	0.02	0.02	0.01	0.09	0.02	0.06	0.01	0.02	0.00	0.12	0.00
Lateral surface	g	4	0.11	0.04	0.02	0.00	0.10	0.05	0.06	0.02	0.02	0.00	0.10	0.04
Lateral surface	h	4	0.10	0.02	0.02	0.01	0.10	0.05	0.07	0.02	0.02	0.00	0.11	0.01
Lateral surface	l	4	0.10	0.14	0.03	0.00	0.12	0.11	0.08	0.01	0.03	0.03	0.07	0.05
Lateral surface	j	3	0.10	0.01	0.01	0.00	0.06	0.00	0.03	0.04	0.01	0.00	0.09	0.03
Lateral surface	k	1	0.06	0.00	0.01	0.00	0.08	0.00	0.13	0.00	0.01	0.00	0.08	0.00
Dorsal condyle		10	0.14	0.04	0.04	0.03	0.03	0.02	0.04	0.01	0.02	0.01	0.06	0.05
Ventral condyle		10	0.00	0.00	0.02	0.00	0.01	0.00	0.01	0.00	0.00	0.00	0.07	0.02

Region	Locality	N	Mn mean	Mean SD	P+Pt, mean	P+Pt, SD	S mean	S SD	Si mean	Si SD	Zn mean	Zn SD
Lateral surface	a	1	0.06	0.00	0.28	0.00	0.08	0.00	0.01	0.00	0.08	0.00
Lateral surface	b	3	0.03	0.02	0.27	0.01	0.04	0.00	0.02	0.00	0.06	0.01
Lateral surface	c	4	0.02	0.00	0.23	0.06	0.02	0.01	0.02	0.01	0.05	0.01
Lateral surface	d	4	0.03	0.00	0.24	0.01	0.03	0.02	0.04	0.04	0.06	0.02
Lateral surface	e	4	0.01	0.01	0.20	0.10	0.01	0.01	0.05	0.07	0.02	0.01
Lateral surface	f	4	0.03	0.01	0.19	0.01	0.02	0.01	0.01	0.01	0.07	0.06
Lateral surface	g	4	0.02	0.00	0.22	0.01	0.03	0.01	0.02	0.01	0.05	0.00
Lateral surface	h	4	0.03	0.01	0.19	0.08	0.02	0.00	0.02	0.02	0.04	0.01
Lateral surface	l	4	0.03	0.02	0.24	0.08	0.02	0.00	0.03	0.03	0.07	0.03
Lateral surface	j	4	0.04	0.02	0.21	0.03	0.03	0.00	0.02	0.01	0.06	0.02
Lateral surface	k	4	0.02	0.00	0.25	0.02	0.03	0.00	0.02	0.00	0.07	0.02
Tip, Medial surface	a	20	0.03	0.01	0.19	0.07	0.03	0.01	0.04	0.03	0.06	0.03
Tip, Lateral surface	a‘	21	0.02	0.01	0.16	0.06	0.02	0.01	0.02	0.01	0.05	0.02
Lateral surface	a	4	0.03	0.03	0.30	0.05	0.03	0.02	0.02	0.01	0.07	0.02
Lateral surface	b	4	0.01	0.01	0.26	0.03	0.03	0.00	0.03	0.00	0.05	0.01
Lateral surface	c	4	0.03	0.02	0.21	0.06	0.03	0.01	0.05	0.07	0.05	0.04
Lateral surface	d	4	0.07	0.05	0.23	0.05	0.09	0.07	0.10	0.18	0.28	0.23
Lateral surface	e	4	0.01	0.01	0.18	0.01	0.03	0.00	0.01	0.01	0.03	0.01
Lateral surface	f	4	0.02	0.00	0.23	0.04	0.02	0.00	0.02	0.00	0.08	0.01
Lateral surface	g	4	0.02	0.00	0.27	0.09	0.02	0.00	0.02	0.00	0.06	0.02
Lateral surface	h	4	0.03	0.01	0.24	0.07	0.02	0.00	0.02	0.00	0.06	0.02
Lateral surface	l	4	0.03	0.02	0.35	0.26	0.03	0.01	0.02	0.00	0.08	0.04
Lateral surface	j	3	0.01	0.00	0.24	0.05	0.02	0.00	0.02	0.00	0.06	0.00
Lateral surface	k	1	0.02	0.00	0.25	0.00	0.03	0.00	0.01	0.00	0.08	0.00
Dorsal condyle		10	0.03	0.02	0.19	0.10	0.02	0.01	0.01	0.01	0.03	0.02
Ventral condyle		10	0.00	0.00	0.10	0.02	0.00	0.00	0.00	0.00	0.01	0.00

Supplementary Table 6. Results from nanoindentation measurements. Hardness *H* and Young's modulus *E*, both given in GPa, sorted according to the tested region. Abbreviations: N, quantity of measurements; SD, standard deviation.

Region	Medial or Lateral surface	Locality	N	*H* Mean	*H* SD	*E* Mean	*E* SD
Tip	Medial	a	8	0.05	0.02	2.20	0.46
Surface	Medial	a	8	0.10	0.02	2.54	0.40
Surface	Medial	b	8	0.13	0.02	2.72	0.55
Surface	Medial	c	8	0.13	0.02	3.20	0.47
Surface	Medial	d	8	0.14	0.03	3.46	0.25
Surface	Medial	e	8	0.19	0.02	4.56	0.52
Surface	Medial	f	8	0.25	0.02	6.07	0.36
Surface	Medial	g	8	0.27	0.03	6.13	0.46
Surface	Medial	h	8	0.28	0.03	6.61	0.46
Surface	Medial	i	8	0.29	0.02	7.15	0.22
Surface	Medial	j	8	0.30	0.03	6.90	0.44
Surface	Medial	k	8	0.33	0.04	7.21	0.56
Tip	Lateral	a'	8	0.24	0.05	6.35	0.21
Surface	Lateral	a	8	0.27	0.03	6.07	0.24
Surface	Lateral	b	8	0.26	0.04	6.32	0.26
Surface	Lateral	c	8	0.27	0.04	6.42	0.21
Surface	Lateral	d	8	0.28	0.03	6.56	0.21
Surface	Lateral	e	8	0.26	0.03	6.38	0.27
Surface	Lateral	f	8	0.27	0.05	6.76	0.22
Surface	Lateral	g	8	0.30	0.04	6.75	0.16
Surface	Lateral	h	8	0.29	0.05	6.94	0.21
Surface	Lateral	i	8	0.32	0.05	7.07	0.17
Surface	Lateral	j	8	0.32	0.05	7.03	0.45
Surface	Lateral	k	8	0.37	0.05	7.41	0.44
Condyle	Dorsal		8	0.37	0.07	7.97	0.25
Condyle	Ventral		8	0.37	0.06	8.06	0.31

Supplementary Table 7. Results from Kruskal-Wallis-test and p-values from pairwise comparison by Wilcoxon method for the hardness data *H*. p-values: p < 0.001 = highly significant differences; 0.05 < p < 0.10 = significant differences; p > 0.10 = no significant differences.

Parameter	Region 1	Region 2	Results from Kruskal-Wallis-test			p-value
			Chi ^{2}	DF	p-value	
H	Surface, Medial b	Tip, Medial surface a	151.4593	25	<.0001*	0.0009*
H	Surface, Medial c	Tip, Medial surface a				0.0009*
H	Surface, Medial d	Tip, Medial surface a				0.0009*
H	Surface, Medial e	Tip, Medial surface a				0.0009*
H	Surface, Medial e	Surface, Medial a				0.0009*
H	Surface, Medial f	Tip, Medial surface a				0.0009*
H	Surface, Medial f	Surface, Medial a				0.0009*
H	Surface, Medial f	Surface, Medial b				0.0009*
H	Surface, Medial f	Surface, Medial c				0.0009*
H	Surface, Medial f	Surface, Medial d				0.0009*
H	Surface, Medial g	Tip, Medial surface a				0.0009*
H	Surface, Medial g	Surface, Medial a				0.0009*
H	Surface, Medial g	Surface, Medial b				0.0009*
H	Surface, Medial g	Surface, Medial c				0.0009*
H	Surface, Medial g	Surface, Medial d				0.0009*
H	Surface, Medial g	Surface, Medial e				0.0009*
H	Surface, Medial h	Tip, Medial surface a				0.0009*
H	Surface, Medial h	Surface, Medial a				0.0009*
H	Surface, Medial h	Surface, Medial b				0.0009*
H	Surface, Medial h	Surface, Medial c				0.0009*
H	Surface, Medial h	Surface, Medial d				0.0009*
H	Surface, Medial h	Surface, Medial e				0.0009*
H	Surface, Medial i	Tip, Medial surface a				0.0009*
H	Surface, Medial i	Surface, Medial a				0.0009*
H	Surface, Medial i	Surface, Medial b				0.0009*
H	Surface, Medial i	Surface, Medial c				0.0009*
H	Surface, Medial i	Surface, Medial d				0.0009*
H	Surface, Medial i	Surface, Medial e				0.0009*
H	Surface, Medial j	Tip, Medial surface a				0.0009*

H	Surface, Medial j	Surface, Medial a		0.0009*
H	Surface, Medial j	Surface, Medial b		0.0009*
H	Surface, Medial j	Surface, Medial c		0.0009*
H	Surface, Medial j	Surface, Medial d		0.0009*
H	Surface, Medial j	Surface, Medial e		0.0009*
H	Surface, Medial k	Tip, Medial surface a		0.0009*
H	Surface, Medial k	Surface, Medial a		0.0009*
H	Surface, Medial k	Surface, Medial b		0.0009*
H	Surface, Medial k	Surface, Medial c		0.0009*
H	Surface, Medial k	Surface, Medial d		0.0009*
H	Surface, Medial k	Surface, Medial e		0.0009*
H	Tip, Lateral surface a	Tip, Medial surface a		0.0009*
H	Tip, Lateral surface a	Surface, Medial a		0.0009*
H	Tip, Lateral surface a	Surface, Medial b		0.0009*
H	Tip, Lateral surface a	Surface, Medial c		0.0009*
H	Tip, Lateral surface a	Surface, Medial d		0.0009*
H	Surface, Lateral a	Tip, Medial surface a		0.0009*
H	Surface, Lateral a	Surface, Medial a		0.0009*
H	Surface, Lateral a	Surface, Medial b		0.0009*
H	Surface, Lateral a	Surface, Medial c		0.0009*
H	Surface, Lateral a	Surface, Medial d		0.0009*
H	Surface, Lateral b	Tip, Medial surface a		0.0009*
H	Surface, Lateral b	Surface, Medial a		0.0009*
H	Surface, Lateral b	Surface, Medial b		0.0009*
H	Surface, Lateral b	Surface, Medial c		0.0009*
H	Surface, Lateral b	Surface, Medial d		0.0009*
H	Surface, Lateral c	Tip, Medial surface a		0.0009*
H	Surface, Lateral c	Surface, Medial a		0.0009*
H	Surface, Lateral c	Surface, Medial b		0.0009*
H	Surface, Lateral c	Surface, Medial c		0.0009*
H	Surface, Lateral c	Surface, Medial d		0.0009*
H	Surface, Lateral c	Surface, Medial e		0.0009*
H	Surface, Lateral d	Tip, Medial surface a		0.0009*
H	Surface, Lateral d	Surface, Medial a		0.0009*
H	Surface, Lateral d	Surface, Medial b		0.0009*
H	Surface, Lateral d	Surface, Medial c		0.0009*
H	Surface, Lateral d	Surface, Medial d		0.0009*
H	Surface, Lateral d	Surface, Medial e		0.0009*
H	Surface, Lateral e	Tip, Medial surface a		0.0009*
H	Surface, Lateral e	Surface, Medial a		0.0009*
H	Surface, Lateral e	Surface, Medial b		0.0009*
H	Surface, Lateral e	Surface, Medial c		0.0009*
H	Surface, Lateral e	Surface, Medial d		0.0009*
H	Surface, Lateral f	Tip, Medial surface a		0.0009*
H	Surface, Lateral f	Surface, Medial a		0.0009*
H	Surface, Lateral f	Surface, Medial b		0.0009*
H	Surface, Lateral f	Surface, Medial c		0.0009*
H	Surface, Lateral f	Surface, Medial d		0.0009*
H	Surface, Lateral g	Tip, Medial surface a		0.0009*
H	Surface, Lateral g	Surface, Medial a		0.0009*
H	Surface, Lateral g	Surface, Medial b		0.0009*
H	Surface, Lateral g	Surface, Medial c		0.0009*
H	Surface, Lateral g	Surface, Medial d		0.0009*
H	Surface, Lateral h	Tip, Medial surface a		0.0009*
H	Surface, Lateral h	Surface, Medial a		0.0009*
H	Surface, Lateral h	Surface, Medial b		0.0009*
H	Surface, Lateral h	Surface, Medial c		0.0009*
H	Surface, Lateral h	Surface, Medial d		0.0009*
H	Surface, Lateral h	Surface, Medial e		0.0009*
H	Surface, Lateral i	Tip, Medial surface a		0.0009*
H	Surface, Lateral i	Surface, Medial a		0.0009*
H	Surface, Lateral i	Surface, Medial b		0.0009*
H	Surface, Lateral i	Surface, Medial c		0.0009*
H	Surface, Lateral i	Surface, Medial d		0.0009*
H	Surface, Lateral i	Surface, Medial e		0.0009*
H	Surface, Lateral j	Tip, Medial surface a		0.0009*
H	Surface, Lateral j	Surface, Medial a		0.0009*
H	Surface, Lateral j	Surface, Medial b		0.0009*

H	Surface, Lateral j	Surface, Medial c	0.0009*
H	Surface, Lateral j	Surface, Medial d	0.0009*
H	Surface, Lateral j	Surface, Medial e	0.0009*
H	Surface, Lateral k	Tip, Medial surface a	0.0009*
H	Surface, Lateral k	Surface, Medial a	0.0009*
H	Surface, Lateral k	Surface, Medial b	0.0009*
H	Surface, Lateral k	Surface, Medial c	0.0009*
H	Surface, Lateral k	Surface, Medial d	0.0009*
H	Surface, Lateral k	Surface, Medial e	0.0009*
H	Surface, Lateral k	Surface, Medial f	0.0009*
H	Dorsal condyle a	Tip, Medial surface a	0.0009*
H	Dorsal condyle a	Surface, Medial a	0.0009*
H	Dorsal condyle a	Surface, Medial b	0.0009*
H	Dorsal condyle a	Surface, Medial c	0.0009*
H	Dorsal condyle a	Surface, Medial d	0.0009*
H	Dorsal condyle a	Surface, Medial e	0.0009*
H	Dorsal condyle a	Surface, Medial f	0.0009*
H	Ventral condyle a	Tip, Medial surface a	0.0009*
H	Ventral condyle a	Surface, Medial a	0.0009*
H	Ventral condyle a	Surface, Medial b	0.0009*
H	Ventral condyle a	Surface, Medial c	0.0009*
H	Ventral condyle a	Surface, Medial d	0.0009*
H	Ventral condyle a	Surface, Medial e	0.0009*
H	Ventral condyle a	Surface, Medial f	0.0009*
H	Surface, Medial f	Surface, Medial e	0.0011*
H	Surface, Medial e	Surface, Medial b	0.0013*
H	Surface, Medial e	Surface, Medial c	0.0014*
H	Surface, Lateral a	Surface, Medial e	0.0014*
H	Surface, Lateral g	Surface, Medial e	0.0013*
H	Surface, Lateral k	Tip, Lateral surface a	0.0013*
H	Surface, Lateral k	Surface, Lateral e	0.0014*
H	Dorsal condyle a	Tip, Lateral surface a	0.0013*
H	Dorsal condyle a	Surface, Lateral e	0.0014*
H	Surface, Medial k	Surface, Medial f	0.0016*
H	Surface, Medial j	Surface, Medial f	0.0019*
H	Ventral condyle a	Tip, Lateral surface a	0.0019*
H	Ventral condyle a	Surface, Lateral e	0.0019*
H	Surface, Lateral j	Surface, Medial f	0.0023*
H	Surface, Medial a	Tip, Medial surface a	0.0028*
H	Surface, Lateral b	Surface, Medial e	0.0028*
H	Surface, Lateral e	Surface, Medial e	0.0028*
H	Surface, Lateral k	Surface, Medial g	0.0028*
H	Surface, Lateral k	Surface, Lateral a	0.0028*
H	Surface, Lateral k	Surface, Lateral b	0.0028*
H	Dorsal condyle a	Surface, Medial g	0.0028*
H	Dorsal condyle a	Surface, Lateral b	0.0028*
H	Ventral condyle a	Surface, Medial g	0.0028*
H	Ventral condyle a	Surface, Lateral a	0.0033*
H	Surface, Lateral k	Surface, Medial h	0.0038*
H	Dorsal condyle a	Surface, Lateral a	0.0039*
H	Ventral condyle a	Surface, Lateral b	0.0039*
H	Surface, Lateral i	Surface, Medial f	0.0045*
H	Surface, Medial e	Surface, Medial d	0.0054*
H	Surface, Medial i	Surface, Medial f	0.0053*
H	Surface, Lateral k	Surface, Lateral d	0.0054*
H	Dorsal condyle a	Surface, Lateral c	0.0054*
H	Ventral condyle a	Surface, Medial h	0.0054*
H	Ventral condyle a	Surface, Lateral c	0.0054*
H	Ventral condyle a	Surface, Lateral d	0.0054*
H	Surface, Lateral k	Surface, Lateral c	0.0074*
H	Surface, Lateral k	Surface, Lateral f	0.0074*
H	Dorsal condyle a	Surface, Medial h	0.0074*
H	Dorsal condyle a	Surface, Lateral d	0.0074*
H	Surface, Medial h	Surface, Medial f	0.0085*
H	Dorsal condyle a	Surface, Lateral f	0.0100*
H	Ventral condyle a	Surface, Lateral f	0.0100*
H	Surface, Lateral f	Surface, Medial e	0.0135*
H	Surface, Lateral k	Surface, Medial i	0.0135*

H	Surface, Lateral k	Surface, Lateral g	0.0135*
H	Surface, Lateral k	Surface, Lateral h	0.0136*
H	Dorsal condyle a	Surface, Medial i	0.0135*
H	Ventral condyle a	Surface, Medial i	0.0135*
H	Surface, Medial b	Surface, Medial a	0.0156*
H	Surface, Medial d	Surface, Medial a	0.0156*
H	Surface, Lateral g	Surface, Medial f	0.0176*
H	Surface, Lateral k	Surface, Medial j	0.0181*
H	Surface, Medial c	Surface, Medial a	0.0239*
H	Surface, Medial k	Surface, Medial g	0.0236*
H	Surface, Lateral g	Tip, Lateral surface a	0.0310*
H	Surface, Lateral i	Tip, Lateral surface a	0.0311*
H	Surface, Lateral j	Surface, Lateral e	0.0313*
H	Surface, Lateral k	Surface, Lateral i	0.0313*
H	Ventral condyle a	Surface, Lateral g	0.0312*
H	Surface, Lateral g	Surface, Lateral e	0.0404*
H	Surface, Lateral i	Surface, Lateral e	0.0406*
H	Surface, Lateral j	Tip, Lateral surface a	0.0403*
H	Surface, Lateral k	Surface, Lateral j	0.0406*
H	Ventral condyle a	Surface, Medial j	0.0406*
H	Surface, Lateral i	Surface, Lateral a	0.0520
H	Surface, Lateral i	Surface, Lateral b	0.0520
H	Surface, Lateral i	Surface, Lateral c	0.0519
H	Dorsal condyle a	Surface, Lateral g	0.0519
H	Ventral condyle a	Surface, Lateral h	0.0520
H	Surface, Medial k	Surface, Medial h	0.0582
H	Surface, Medial j	Surface, Medial g	0.0653
H	Surface, Lateral d	Surface, Medial f	0.0657
H	Surface, Lateral j	Surface, Lateral a	0.0661
H	Surface, Lateral j	Surface, Lateral b	0.0661
H	Dorsal condyle a	Surface, Medial j	0.0661
H	Dorsal condyle a	Surface, Lateral h	0.0661
H	Surface, Lateral h	Surface, Medial f	0.0736
H	Surface, Lateral f	Tip, Lateral surface a	0.0824
H	Surface, Lateral i	Surface, Lateral f	0.0829
H	Surface, Lateral k	Surface, Medial k	0.0829
H	Surface, Lateral i	Surface, Medial g	0.0927
H	Surface, Medial k	Surface, Medial i	0.1025
H	Tip, Lateral surface a	Surface, Medial e	0.1031
H	Surface, Lateral g	Surface, Lateral b	0.1033
H	Surface, Lateral h	Tip, Lateral surface a	0.1031
H	Surface, Lateral i	Surface, Lateral d	0.1036
H	Surface, Lateral j	Surface, Medial g	0.1031
H	Surface, Lateral j	Surface, Lateral c	0.1033
H	Dorsal condyle a	Surface, Lateral j	0.1036
H	Ventral condyle a	Surface, Lateral j	0.1036
H	Surface, Medial g	Surface, Medial f	0.1144
H	Surface, Lateral d	Tip, Lateral surface a	0.1272
H	Surface, Lateral c	Tip, Lateral surface a	0.1553
H	Surface, Lateral g	Surface, Lateral a	0.1559
H	Surface, Lateral j	Surface, Lateral d	0.1563
H	Surface, Medial i	Surface, Medial g	0.1715
H	Surface, Lateral g	Surface, Medial g	0.1706
H	Surface, Medial j	Surface, Medial h	0.1876
H	Surface, Lateral a	Surface, Medial f	0.1886
H	Surface, Lateral j	Surface, Lateral f	0.1889
H	Dorsal condyle a	Surface, Lateral i	0.1893
H	Ventral condyle a	Surface, Lateral i	0.1893
H	Surface, Lateral j	Surface, Medial h	0.2069
H	Surface, Medial k	Surface, Medial j	0.2254
H	Surface, Lateral h	Surface, Lateral e	0.2271
H	Surface, Lateral i	Surface, Medial h	0.2268
H	Surface, Lateral j	Surface, Lateral h	0.2271
H	Ventral condyle a	Surface, Medial k	0.2268
H	Surface, Lateral c	Surface, Medial f	0.2691
H	Surface, Lateral g	Surface, Lateral d	0.2698
H	Surface, Lateral i	Surface, Medial i	0.2698
H	Dorsal condyle a	Surface, Medial k	0.2698

H	Surface, Lateral h	Surface, Lateral b	0.2933
H	Surface, Lateral i	Surface, Lateral h	0.2933
H	Surface, Medial d	Surface, Medial b	0.3174
H	Surface, Lateral b	Surface, Medial f	0.3177
H	Surface, Lateral d	Surface, Lateral b	0.3184
H	Surface, Lateral g	Surface, Lateral c	0.3177
H	Surface, Lateral g	Surface, Lateral f	0.3177
H	Surface, Lateral h	Surface, Lateral f	0.3181
H	Surface, Lateral i	Surface, Lateral g	0.3170
H	Surface, Lateral e	Surface, Medial f	0.3713
H	Surface, Lateral h	Surface, Lateral c	0.3717
H	Surface, Medial i	Surface, Medial h	0.3991
H	Surface, Lateral h	Surface, Lateral a	0.4005
H	Surface, Lateral a	Tip, Lateral surface a	0.4302
H	Surface, Lateral f	Surface, Medial f	0.4299
H	Surface, Lateral g	Surface, Medial h	0.4295
H	Surface, Lateral i	Surface, Medial j	0.4619
H	Surface, Medial d	Surface, Medial c	0.4948
H	Surface, Medial j	Surface, Medial i	0.4939
H	Surface, Lateral d	Surface, Lateral a	0.4948
H	Surface, Lateral e	Tip, Lateral surface a	0.4942
H	Surface, Lateral f	Surface, Lateral e	0.4929
H	Surface, Lateral h	Surface, Medial g	0.4948
H	Surface, Lateral j	Surface, Lateral g	0.4929
H	Surface, Medial h	Surface, Medial g	0.5627
H	Surface, Lateral b	Tip, Lateral surface a	0.5629
H	Surface, Lateral c	Surface, Lateral b	0.5632
H	Surface, Lateral j	Surface, Medial i	0.5632
H	Surface, Lateral j	Surface, Medial j	0.5987
H	Surface, Lateral d	Surface, Lateral c	0.6358
H	Surface, Lateral f	Surface, Lateral b	0.6363
H	Surface, Lateral h	Surface, Lateral d	0.6365
H	Dorsal condyle a	Surface, Lateral k	0.6360
H	Surface, Lateral d	Surface, Medial g	0.7132
H	Surface, Medial c	Surface, Medial b	0.7524
H	Surface, Lateral g	Surface, Medial i	0.8333
H	Surface, Lateral j	Surface, Lateral i	0.8335
H	Surface, Lateral c	Surface, Lateral a	0.8747
H	Surface, Lateral f	Surface, Medial g	0.8747
H	Surface, Lateral c	Surface, Medial g	0.9581
H	Surface, Lateral f	Surface, Lateral a	0.9581
H	Ventral condyle a	Surface, Lateral k	0.9581
H	Surface, Lateral h	Surface, Medial h	1.0000
H	Surface, Lateral h	Surface, Medial i	1.0000
H	Tip, Lateral surface a	Surface, Medial f	0.9580
H	Surface, Lateral f	Surface, Medial h	0.9581
H	Surface, Lateral a	Surface, Medial g	0.8748
H	Surface, Lateral d	Surface, Medial h	0.8747
H	Surface, Lateral e	Surface, Lateral b	0.8748
H	Surface, Lateral f	Surface, Lateral d	0.8747
H	Ventral condyle a	Dorsal condyle a	0.8746
H	Surface, Lateral b	Surface, Lateral a	0.8333
H	Surface, Lateral h	Surface, Lateral g	0.8334
H	Surface, Lateral e	Surface, Lateral a	0.7929
H	Surface, Lateral f	Surface, Lateral c	0.7926
H	Surface, Lateral g	Surface, Medial j	0.7917
H	Surface, Lateral b	Surface, Medial g	0.7132
H	Surface, Lateral i	Surface, Medial k	0.6740
H	Surface, Lateral j	Surface, Medial k	0.6735
H	Surface, Lateral c	Surface, Medial h	0.6360
H	Surface, Lateral e	Surface, Lateral c	0.6363
H	Surface, Lateral h	Surface, Medial j	0.5992
H	Surface, Lateral e	Surface, Medial g	0.4948
H	Surface, Lateral f	Surface, Medial i	0.4942
H	Surface, Lateral c	Surface, Medial i	0.4302
H	Surface, Lateral d	Surface, Medial i	0.4306
H	Surface, Lateral a	Surface, Medial h	0.3717
H	Surface, Lateral e	Surface, Lateral d	0.3720

H	Surface, Lateral b	Surface, Medial h				0.3181
H	Tip, Lateral surface a	Surface, Medial g				0.2694
H	Surface, Lateral f	Surface, Medial j				0.2268
H	Tip, Lateral surface a	Surface, Medial h				0.1883
H	Surface, Lateral g	Surface, Medial k				0.1870
H	Surface, Lateral a	Surface, Medial i				0.1275
H	Surface, Lateral b	Surface, Medial i				0.1275
H	Surface, Lateral d	Surface, Medial j				0.1278
H	Surface, Lateral e	Surface, Medial h				0.1275
H	Surface, Lateral h	Surface, Medial k				0.1146
H	Surface, Lateral c	Surface, Medial j				0.1033
H	Surface, Lateral a	Surface, Medial j				0.0520
H	Surface, Lateral f	Surface, Medial k				0.0517
H	Tip, Lateral surface a	Surface, Medial i				0.0401*
H	Surface, Lateral b	Surface, Medial j				0.0406*
H	Surface, Lateral d	Surface, Medial k				0.0312*
H	Surface, Lateral e	Surface, Medial i				0.0312*
H	Surface, Lateral c	Surface, Medial k				0.0237*
H	Tip, Lateral surface a	Surface, Medial j				0.0180*
H	Surface, Lateral b	Surface, Medial k				0.0180*
H	Tip, Lateral surface a	Surface, Medial k				0.0134*
H	Surface, Lateral a	Surface, Medial k				0.0135*
H	Surface, Lateral e	Surface, Medial j				0.0136*
H	Surface, Lateral e	Surface, Medial k				0.0074*

Supplementary Table 8. Results from Kruskal-Wallis-test and p-values from pairwise comparison by Wilcoxon method for the Young's modulus *E*. p-values: $p < 0.001$ = highly significant differences; $0.05 < p < 0.10$ = significant differences; $p > 0.10$ = no significant differences.

Parameter	Region 1	Region 2	Results from Kruskal-Wallis-test			p-value
			Chi ^{2}	DF	p-value	
E	Surface, Medial e	Tip, Medial surface a	181.3650	25	<.0001*	0.0009*
E	Surface, Medial e	Surface, Medial a				0.0009*
E	Surface, Medial e	Surface, Medial b				0.0009*
E	Surface, Medial f	Tip, Medial surface a				0.0009*
E	Surface, Medial f	Surface, Medial a				0.0009*
E	Surface, Medial f	Surface, Medial b				0.0009*
E	Surface, Medial f	Surface, Medial c				0.0009*
E	Surface, Medial f	Surface, Medial d				0.0009*
E	Surface, Medial f	Surface, Medial e				0.0009*
E	Surface, Medial g	Tip, Medial surface a				0.0009*
E	Surface, Medial g	Surface, Medial a				0.0009*
E	Surface, Medial g	Surface, Medial b				0.0009*
E	Surface, Medial g	Surface, Medial c				0.0009*
E	Surface, Medial g	Surface, Medial d				0.0009*
E	Surface, Medial g	Surface, Medial e				0.0009*
E	Surface, Medial h	Tip, Medial surface a				0.0009*
E	Surface, Medial h	Surface, Medial a				0.0009*
E	Surface, Medial h	Surface, Medial b				0.0009*
E	Surface, Medial h	Surface, Medial c				0.0009*
E	Surface, Medial h	Surface, Medial d				0.0009*
E	Surface, Medial h	Surface, Medial e				0.0009*
E	Surface, Medial i	Tip, Medial surface a				0.0009*
E	Surface, Medial i	Surface, Medial a				0.0009*
E	Surface, Medial i	Surface, Medial b				0.0009*
E	Surface, Medial i	Surface, Medial c				0.0009*
E	Surface, Medial i	Surface, Medial d				0.0009*
E	Surface, Medial i	Surface, Medial e				0.0009*
E	Surface, Medial j	Tip, Medial surface a				0.0009*
E	Surface, Medial j	Surface, Medial a				0.0009*
E	Surface, Medial j	Surface, Medial b				0.0009*
E	Surface, Medial j	Surface, Medial c				0.0009*
E	Surface, Medial j	Surface, Medial d				0.0009*
E	Surface, Medial j	Surface, Medial e				0.0009*
E	Surface, Medial k	Tip, Medial surface a				0.0009*
E	Surface, Medial k	Surface, Medial a				0.0009*
E	Surface, Medial k	Surface, Medial b				0.0009*
E	Surface, Medial k	Surface, Medial c				0.0009*
E	Surface, Medial k	Surface, Medial d				0.0009*

E	Surface, Medial k	Surface, Medial e		0.0009*
E	Tip, Lateral surface a	Tip, Medial surface a		0.0009*
E	Tip, Lateral surface a	Surface, Medial a		0.0009*
E	Tip, Lateral surface a	Surface, Medial b		0.0009*
E	Tip, Lateral surface a	Surface, Medial c		0.0009*
E	Tip, Lateral surface a	Surface, Medial d		0.0009*
E	Tip, Lateral surface a	Surface, Medial e		0.0009*
E	Surface, Lateral a	Tip, Medial surface a		0.0009*
E	Surface, Lateral a	Surface, Medial a		0.0009*
E	Surface, Lateral a	Surface, Medial b		0.0009*
E	Surface, Lateral a	Surface, Medial c		0.0009*
E	Surface, Lateral a	Surface, Medial d		0.0009*
E	Surface, Lateral a	Surface, Medial e		0.0009*
E	Surface, Lateral b	Tip, Medial surface a		0.0009*
E	Surface, Lateral b	Surface, Medial a		0.0009*
E	Surface, Lateral b	Surface, Medial b		0.0009*
E	Surface, Lateral b	Surface, Medial c		0.0009*
E	Surface, Lateral b	Surface, Medial d		0.0009*
E	Surface, Lateral b	Surface, Medial e		0.0009*
E	Surface, Lateral c	Tip, Medial surface a		0.0009*
E	Surface, Lateral c	Surface, Medial a		0.0009*
E	Surface, Lateral c	Surface, Medial b		0.0009*
E	Surface, Lateral c	Surface, Medial c		0.0009*
E	Surface, Lateral c	Surface, Medial d		0.0009*
E	Surface, Lateral c	Surface, Medial e		0.0009*
E	Surface, Lateral d	Tip, Medial surface a		0.0009*
E	Surface, Lateral d	Surface, Medial a		0.0009*
E	Surface, Lateral d	Surface, Medial b		0.0009*
E	Surface, Lateral d	Surface, Medial c		0.0009*
E	Surface, Lateral d	Surface, Medial d		0.0009*
E	Surface, Lateral d	Surface, Medial e		0.0009*
E	Surface, Lateral e	Tip, Medial surface a		0.0009*
E	Surface, Lateral e	Surface, Medial a		0.0009*
E	Surface, Lateral e	Surface, Medial b		0.0009*
E	Surface, Lateral e	Surface, Medial c		0.0009*
E	Surface, Lateral e	Surface, Medial d		0.0009*
E	Surface, Lateral e	Surface, Medial e		0.0009*
E	Surface, Lateral f	Tip, Medial surface a		0.0009*
E	Surface, Lateral f	Surface, Medial a		0.0009*
E	Surface, Lateral f	Surface, Medial b		0.0009*
E	Surface, Lateral f	Surface, Medial c		0.0009*
E	Surface, Lateral f	Surface, Medial d		0.0009*
E	Surface, Lateral f	Surface, Medial e		0.0009*
E	Surface, Lateral f	Surface, Lateral a		0.0009*
E	Surface, Lateral g	Tip, Medial surface a		0.0009*
E	Surface, Lateral g	Surface, Medial a		0.0009*
E	Surface, Lateral g	Surface, Medial b		0.0009*
E	Surface, Lateral g	Surface, Medial c		0.0009*
E	Surface, Lateral g	Surface, Medial d		0.0009*
E	Surface, Lateral g	Surface, Medial e		0.0009*
E	Surface, Lateral g	Surface, Lateral a		0.0009*
E	Surface, Lateral h	Tip, Medial surface a		0.0009*
E	Surface, Lateral h	Surface, Medial a		0.0009*
E	Surface, Lateral h	Surface, Medial b		0.0009*
E	Surface, Lateral h	Surface, Medial c		0.0009*
E	Surface, Lateral h	Surface, Medial d		0.0009*
E	Surface, Lateral h	Surface, Medial e		0.0009*
E	Surface, Lateral h	Tip, Lateral surface a		0.0009*
E	Surface, Lateral h	Surface, Lateral a		0.0009*
E	Surface, Lateral i	Tip, Medial surface a		0.0009*
E	Surface, Lateral i	Surface, Medial a		0.0009*
E	Surface, Lateral i	Surface, Medial b		0.0009*
E	Surface, Lateral i	Surface, Medial c		0.0009*
E	Surface, Lateral i	Surface, Medial d		0.0009*
E	Surface, Lateral i	Surface, Medial e		0.0009*
E	Surface, Lateral i	Surface, Medial f		0.0009*
E	Surface, Lateral i	Surface, Medial g		0.0009*
E	Surface, Lateral i	Tip, Lateral surface a		0.0009*

E	Surface, Lateral i	Surface, Lateral a	0.0009*
E	Surface, Lateral i	Surface, Lateral b	0.0009*
E	Surface, Lateral i	Surface, Lateral c	0.0009*
E	Surface, Lateral i	Surface, Lateral d	0.0009*
E	Surface, Lateral i	Surface, Lateral e	0.0009*
E	Surface, Lateral j	Tip, Medial surface a	0.0009*
E	Surface, Lateral j	Surface, Medial a	0.0009*
E	Surface, Lateral j	Surface, Medial b	0.0009*
E	Surface, Lateral j	Surface, Medial c	0.0009*
E	Surface, Lateral j	Surface, Medial d	0.0009*
E	Surface, Lateral j	Surface, Medial e	0.0009*
E	Surface, Lateral k	Tip, Medial surface a	0.0009*
E	Surface, Lateral k	Surface, Medial a	0.0009*
E	Surface, Lateral k	Surface, Medial b	0.0009*
E	Surface, Lateral k	Surface, Medial c	0.0009*
E	Surface, Lateral k	Surface, Medial d	0.0009*
E	Surface, Lateral k	Surface, Medial e	0.0009*
E	Surface, Lateral k	Surface, Lateral a	0.0009*
E	Dorsal condyle a	Tip, Medial surface a	0.0009*
E	Dorsal condyle a	Surface, Medial a	0.0009*
E	Dorsal condyle a	Surface, Medial b	0.0009*
E	Dorsal condyle a	Surface, Medial c	0.0009*
E	Dorsal condyle a	Surface, Medial d	0.0009*
E	Dorsal condyle a	Surface, Medial e	0.0009*
E	Dorsal condyle a	Surface, Medial f	0.0009*
E	Dorsal condyle a	Surface, Medial g	0.0009*
E	Dorsal condyle a	Surface, Medial i	0.0009*
E	Dorsal condyle a	Tip, Lateral surface a	0.0009*
E	Dorsal condyle a	Surface, Lateral a	0.0009*
E	Dorsal condyle a	Surface, Lateral b	0.0009*
E	Dorsal condyle a	Surface, Lateral c	0.0009*
E	Dorsal condyle a	Surface, Lateral d	0.0009*
E	Dorsal condyle a	Surface, Lateral e	0.0009*
E	Dorsal condyle a	Surface, Lateral f	0.0009*
E	Dorsal condyle a	Surface, Lateral g	0.0009*
E	Dorsal condyle a	Surface, Lateral h	0.0009*
E	Dorsal condyle a	Surface, Lateral i	0.0009*
E	Dorsal condyle a	Surface, Lateral j	0.0009*
E	Ventral condyle a	Tip, Medial surface a	0.0009*
E	Ventral condyle a	Surface, Medial a	0.0009*
E	Ventral condyle a	Surface, Medial b	0.0009*
E	Ventral condyle a	Surface, Medial c	0.0009*
E	Ventral condyle a	Surface, Medial d	0.0009*
E	Ventral condyle a	Surface, Medial e	0.0009*
E	Ventral condyle a	Surface, Medial f	0.0009*
E	Ventral condyle a	Surface, Medial g	0.0009*
E	Ventral condyle a	Surface, Medial i	0.0009*
E	Ventral condyle a	Tip, Lateral surface a	0.0009*
E	Ventral condyle a	Surface, Lateral a	0.0009*
E	Ventral condyle a	Surface, Lateral b	0.0009*
E	Ventral condyle a	Surface, Lateral c	0.0009*
E	Ventral condyle a	Surface, Lateral d	0.0009*
E	Ventral condyle a	Surface, Lateral e	0.0009*
E	Ventral condyle a	Surface, Lateral f	0.0009*
E	Ventral condyle a	Surface, Lateral g	0.0009*
E	Ventral condyle a	Surface, Lateral h	0.0009*
E	Ventral condyle a	Surface, Lateral i	0.0009*
E	Ventral condyle a	Surface, Lateral j	0.0009*
E	Surface, Medial d	Tip, Medial surface a	0.0014*
E	Surface, Medial d	Surface, Medial a	0.0014*
E	Surface, Medial i	Surface, Medial f	0.0014*
E	Surface, Lateral k	Surface, Medial f	0.0014*
E	Dorsal condyle a	Surface, Medial h	0.0014*
E	Dorsal condyle a	Surface, Medial j	0.0014*
E	Ventral condyle a	Surface, Medial h	0.0014*
E	Ventral condyle a	Surface, Medial j	0.0014*
E	Surface, Medial i	Surface, Medial g	0.0019*
E	Surface, Medial k	Surface, Medial f	0.0019*

E	Surface, Medial k	Surface, Medial g	0.0019*
E	Surface, Lateral h	Surface, Lateral b	0.0019*
E	Surface, Lateral j	Surface, Lateral a	0.0019*
E	Surface, Lateral k	Surface, Medial g	0.0019*
E	Surface, Lateral k	Surface, Lateral b	0.0019*
E	Surface, Lateral d	Surface, Lateral a	0.0028*
E	Surface, Lateral g	Tip, Lateral surface a	0.0028*
E	Surface, Lateral h	Surface, Medial f	0.0028*
E	Surface, Lateral h	Surface, Lateral c	0.0028*
E	Surface, Lateral h	Surface, Lateral e	0.0028*
E	Surface, Lateral j	Surface, Medial f	0.0028*
E	Surface, Lateral k	Tip, Lateral surface a	0.0028*
E	Surface, Lateral k	Surface, Lateral c	0.0028*
E	Surface, Lateral k	Surface, Lateral e	0.0028*
E	Surface, Medial e	Surface, Medial c	0.0039*
E	Surface, Lateral i	Surface, Lateral g	0.0039*
E	Surface, Lateral j	Surface, Medial g	0.0039*
E	Ventral condyle a	Surface, Lateral k	0.0039*
E	Surface, Medial c	Tip, Medial surface a	0.0054*
E	Surface, Medial d	Surface, Medial b	0.0054*
E	Surface, Lateral f	Surface, Medial f	0.0054*
E	Surface, Lateral f	Tip, Lateral surface a	0.0054*
E	Surface, Lateral g	Surface, Medial f	0.0054*
E	Surface, Lateral g	Surface, Lateral b	0.0054*
E	Surface, Lateral g	Surface, Lateral c	0.0054*
E	Ventral condyle a	Surface, Medial k	0.0054*
E	Surface, Medial j	Surface, Medial f	0.0074*
E	Surface, Lateral f	Surface, Lateral b	0.0074*
E	Surface, Lateral h	Surface, Medial g	0.0074*
E	Surface, Lateral h	Surface, Lateral d	0.0074*
E	Surface, Lateral j	Surface, Lateral b	0.0074*
E	Surface, Lateral k	Surface, Lateral d	0.0074*
E	Dorsal condyle a	Surface, Lateral k	0.0074*
E	Surface, Medial c	Surface, Medial a	0.0101*
E	Surface, Medial e	Surface, Medial d	0.0101*
E	Surface, Lateral d	Surface, Medial f	0.0101*
E	Surface, Lateral j	Surface, Lateral e	0.0101*
E	Surface, Lateral k	Surface, Lateral f	0.0101*
E	Dorsal condyle a	Surface, Medial k	0.0101*
E	Surface, Medial h	Surface, Medial f	0.0136*
E	Surface, Medial i	Surface, Medial h	0.0136*
E	Surface, Medial j	Surface, Medial g	0.0136*
E	Surface, Lateral f	Surface, Lateral c	0.0136*
E	Surface, Lateral f	Surface, Lateral e	0.0136*
E	Surface, Lateral g	Surface, Lateral e	0.0136*
E	Surface, Lateral i	Surface, Medial h	0.0136*
E	Surface, Lateral i	Surface, Lateral f	0.0136*
E	Surface, Lateral j	Tip, Lateral surface a	0.0136*
E	Surface, Lateral k	Surface, Medial h	0.0136*
E	Surface, Lateral k	Surface, Lateral g	0.0136*
E	Surface, Medial c	Surface, Medial b	0.0181*
E	Surface, Lateral c	Surface, Lateral a	0.0181*
E	Surface, Lateral j	Surface, Lateral c	0.0181*
E	Surface, Lateral k	Surface, Lateral h	0.0181*
E	Surface, Lateral f	Surface, Medial g	0.0239*
E	Surface, Lateral c	Surface, Medial f	0.0313*
E	Surface, Lateral e	Surface, Lateral a	0.0313*
E	Surface, Lateral g	Surface, Medial g	0.0313*
E	Surface, Lateral h	Surface, Medial h	0.0313*
E	Surface, Lateral k	Surface, Medial j	0.0313*
E	Surface, Lateral k	Surface, Lateral i	0.0313*
E	Tip, Lateral surface a	Surface, Medial f	0.0406*
E	Surface, Lateral e	Surface, Medial f	0.0406*
E	Surface, Lateral j	Surface, Lateral d	0.0406*
E	Surface, Medial k	Surface, Medial h	0.0520
E	Surface, Lateral k	Surface, Lateral j	0.0520
E	Surface, Medial b	Tip, Medial surface a	0.0661
E	Surface, Medial h	Surface, Medial g	0.0661

E	Surface, Lateral d	Surface, Medial g		0.0661
E	Surface, Lateral d	Surface, Lateral b		0.0661
E	Surface, Lateral k	Surface, Medial i		0.0661
E	Surface, Medial a	Tip, Medial surface a		0.0831
E	Surface, Medial k	Surface, Medial j		0.0831
E	Surface, Lateral b	Surface, Medial f		0.0831
E	Surface, Lateral g	Surface, Medial h		0.0831
E	Surface, Medial d	Surface, Medial c		0.1036
E	Surface, Medial j	Surface, Medial h		0.1036
E	Surface, Lateral b	Surface, Lateral a		0.1036
E	Surface, Lateral d	Tip, Lateral surface a		0.1036
E	Surface, Lateral e	Surface, Medial g		0.1036
E	Surface, Lateral f	Surface, Lateral d		0.1036
E	Surface, Lateral h	Surface, Lateral f		0.1036
E	Surface, Lateral j	Surface, Medial h		0.1036
E	Surface, Lateral j	Surface, Lateral g		0.1036
E	Surface, Lateral g	Surface, Lateral d		0.1278
E	Surface, Lateral h	Surface, Lateral g		0.1278
E	Surface, Lateral j	Surface, Lateral f		0.1278
E	Surface, Medial b	Surface, Medial a		0.1563
E	Tip, Lateral surface a	Surface, Medial g		0.1563
E	Surface, Lateral c	Surface, Medial g		0.1563
E	Surface, Lateral f	Surface, Medial h		0.1563
E	Surface, Lateral i	Surface, Medial j		0.1563
E	Surface, Lateral b	Surface, Medial g		0.1893
E	Surface, Lateral d	Surface, Lateral c		0.2701
E	Surface, Lateral j	Surface, Lateral h		0.2701
E	Surface, Lateral i	Surface, Lateral h		0.3184
E	Surface, Lateral j	Surface, Medial j		0.3184
E	Surface, Lateral k	Surface, Medial k		0.3184
E	Surface, Lateral c	Tip, Lateral surface a		0.4948
E	Surface, Lateral c	Surface, Lateral b		0.4948
E	Surface, Lateral d	Surface, Medial h		0.5635
E	Ventral condyle a	Dorsal condyle a		0.5635
E	Surface, Lateral e	Surface, Lateral b		0.6365
E	Surface, Lateral a	Surface, Medial f		0.7132
E	Surface, Lateral a	Surface, Medial g		0.7132
E	Surface, Lateral j	Surface, Lateral i		0.7132
E	Surface, Medial k	Surface, Medial i		0.7929
E	Surface, Lateral h	Surface, Medial j		0.7929
E	Surface, Lateral j	Surface, Medial i		1.0000
E	Surface, Lateral e	Tip, Lateral surface a		0.9581
E	Surface, Lateral g	Surface, Lateral f		0.9581
E	Surface, Medial g	Surface, Medial f		0.8748
E	Surface, Lateral j	Surface, Medial k		0.8748
E	Surface, Lateral e	Surface, Lateral c		0.7132
E	Surface, Lateral b	Tip, Lateral surface a		0.6365
E	Surface, Lateral c	Surface, Medial h		0.6365
E	Surface, Lateral i	Surface, Medial i		0.5635
E	Tip, Lateral surface a	Surface, Medial h		0.3184
E	Surface, Lateral e	Surface, Medial h		0.3184
E	Surface, Lateral f	Surface, Medial j		0.3184
E	Surface, Lateral i	Surface, Medial k		0.3184
E	Surface, Lateral b	Surface, Medial h		0.1563
E	Surface, Lateral g	Surface, Medial j		0.1563
E	Surface, Lateral h	Surface, Medial k		0.1563
E	Surface, Lateral e	Surface, Lateral d		0.1278
E	Surface, Medial j	Surface, Medial i		0.0831
E	Surface, Lateral h	Surface, Medial i		0.0661
E	Surface, Lateral a	Tip, Lateral surface a		0.0406*
E	Surface, Lateral d	Surface, Medial j		0.0406*
E	Surface, Lateral f	Surface, Medial k		0.0313*
E	Surface, Lateral e	Surface, Medial j		0.0239*
E	Surface, Lateral g	Surface, Medial k		0.0181*
E	Surface, Lateral b	Surface, Medial j		0.0136*
E	Surface, Lateral d	Surface, Medial k		0.0136*
E	Tip, Lateral surface a	Surface, Medial j		0.0101*
E	Surface, Lateral c	Surface, Medial j		0.0101*

E	Surface, Lateral c	Surface, Medial k		0.0101*
E	Tip, Lateral surface a	Surface, Medial k		0.0074*
E	Surface, Lateral e	Surface, Medial k		0.0074*
E	Surface, Lateral f	Surface, Medial i		0.0074*
E	Surface, Lateral a	Surface, Medial h		0.0054*
E	Surface, Lateral a	Surface, Medial j		0.0054*
E	Surface, Lateral b	Surface, Medial k		0.0054*
E	Surface, Lateral g	Surface, Medial i		0.0054*
E	Surface, Lateral a	Surface, Medial k		0.0019*
E	Surface, Lateral d	Surface, Medial i		0.0014*
E	Surface, Lateral e	Surface, Medial i		0.0014*
E	Tip, Lateral surface a	Surface, Medial i		0.0009*
E	Surface, Lateral a	Surface, Medial i		0.0009*
E	Surface, Lateral b	Surface, Medial i		0.0009*
E	Surface, Lateral c	Surface, Medial i		0.0009*

Supplementary Table 9. P-values from pairwise comparison by Wilcoxon method between left and right mandible of each specimen for the results from nanoindentation, sorted to the region. p-values: $p < 0.001$ = highly significant differences; $0.05 < p < 0.10$ = significant differences; $p > 0.10$ = no significant differences.

Parameter	Specimen	Structure	Side 1	Side 2	p-value
H	C	Mandible tip, lateral surface	Right	Left	1.0000
H	C	Mandible tip, medial surface	Right	Left	1.0000
H	C	Condyle, dorsal	Right	Left	1.0000
H	C	Condyle, ventral	Right	Left	1.0000
H	C	Mandible, medial surface	Right	Left	0.8955
H	C	Mandible, lateral surface	Right	Left	0.3244
E	C	Mandible tip, lateral surface	Right	Left	1.0000
E	C	Mandible tip, medial surface	Right	Left	1.0000
E	C	Condyle, dorsal	Right	Left	1.0000
E	C	Condyle, ventral	Right	Left	1.0000
E	C	Mandible, medial surface	Right	Left	0.6936
E	C	Mandible, lateral surface	Right	Left	0.6458
H	D	Mandible, medial surface	Right	Left	0.8954
H	D	Mandible tip, lateral surface	Right	Left	1.0000
H	D	Mandible tip, medial surface	Right	Left	1.0000
H	D	Condyle, dorsal	Right	Left	1.0000
H	D	Condyle, ventral	Right	Left	1.0000
H	D	Mandible, lateral surface	Right	Left	0.1006
E	D	Mandible tip, lateral surface	Right	Left	1.0000
E	D	Mandible tip, medial surface	Right	Left	1.0000
E	D	Condyle, dorsal	Right	Left	1.0000
E	D	Condyle, ventral	Right	Left	1.0000
E	D	Mandible, lateral surface	Right	Left	0.2372
E	D	Mandible, medial surface	Right	Left	0.9476
H	E	Mandible tip, lateral surface	Right	Left	1.0000
H	E	Mandible tip, medial surface	Right	Left	1.0000
H	E	Condyle, dorsal	Right	Left	1.0000
H	E	Condyle, ventral	Right	Left	1.0000
H	E	Mandible, medial surface	Right	Left	0.3409
H	E	Mandible, lateral surface	Right	Left	0.3085
E	E	Mandible, lateral surface	Right	Left	0.7427
E	E	Mandible tip, lateral surface	Right	Left	1.0000
E	E	Mandible tip, medial surface	Right	Left	1.0000
E	E	Condyle, dorsal	Right	Left	1.0000
E	E	Condyle, ventral	Right	Left	1.0000
E	E	Mandible tip, medial surface	Right	Left	0.6458

Supplementary Table 10. Correlation coefficients estimated by row-wise method are shown for the presence of individual elements (EDX) and the mechanical parameters (nanoindentation).
Correlation coefficients: 1—0.5= moderate to very high correlation; 0.5—0.0= negligible to low correlation.

	**Ca**	**Cl**	**Cu**	**E**	**Fe**	**H**	**K**	**Mg**	**Mn**	**P+Pt**	**S**	**Si**	**Zn**
**Ca**	1.00	0.04	0.66	-0.25	0.67	-0.22	0.56	-0.18	0.74	0.48	0.76	0.03	0.60
**Cl**	-	1.00	0.21	0.08	0.21	0.06	0.70	-0.03	0.17	0.15	0.10	0.21	0.19
**Cu**	-	-	1.00	-0.15	0.85	-0.12	0.61	-0.16	0.76	0.45	0.69	0.09	0.90
**E**	-	-	-	1.00	-0.17	0.90	-0.06	-0.18	-0.06	-0.09	-0.35	-0.19	-0.18
**Fe**	-	-	-	-	1.00	-0.15	0.57	-0.18	0.78	0.31	0.79	0.04	0.87
**H**	-	-	-	-	-	1.00	-0.09	-0.12	-0.07	-0.02	-0.30	-0.15	-0.12
**K**	-	-	-	-	-	-	1.00	-0.02	0.55	0.52	0.50	0.15	0.49
**Mg**	-	-	-		-	-	-	1.00	-0.18	-0.12	-0.10	0.20	-0.16
**Mn**	-	-	-	-	-	-	-	-	1.00	0.35	0.67	0.02	0.77
**P+Pt**	-	-	-	-	-	-	-	-	-	1.00	0.21	0.14	0.25
**S**	-	-	-	-	-	-	-	-	-	-	1.00	0.03	0.78
**Si**	-	-	-	-	-	-	-	-	-	-	-	1.00	0.06
**Zn**	-	-	-	-	-	-	-	-	-	-	-	-	1.00
